# Multi‐omics analysis reveals a crosstalk between ferroptosis and peroxisomes on steatotic graft failure after liver transplantation

**DOI:** 10.1002/mco2.588

**Published:** 2024-06-12

**Authors:** Zhengtao Liu, Hai Zhu, Junsheng Zhao, Lu Yu, Shuping Que, Jun Xu, Lei Geng, Lin Zhou, Luca Valenti, Shusen Zheng

**Affiliations:** ^1^ Shulan International Medical College Zhejiang Shuren University Hangzhou China; ^2^ Key Laboratory of Artificial Organs and Computational Medicine in Zhejiang Province Shulan International Medical College Zhejiang Shuren University Hangzhou China; ^3^ NHC Key Laboratory of Combined Multi‐Organ Transplantation Key Laboratory of the Diagnosis and Treatment of Organ Transplantation CAMS, First Affiliated Hospital School of Medicine Zhejiang University Hangzhou China; ^4^ Key Laboratory of Organ Transplantation First Affiliated Hospital School of Medicine Zhejiang University Hangzhou China; ^5^ Shulan Hospital (Hangzhou) Hangzhou China; ^6^ Department of Hepatobiliary Surgery First Affiliated Hospital of Guangxi Medical University Nanning China; ^7^ School of Medicine Zhejiang Chinese Medical University Hangzhou China; ^8^ DingXiang Clinics Hangzhou China; ^9^ Division of Hepatobiliary and Pancreatic Surgery Department of Surgery First Affiliated Hospital School of Medicine Zhejiang University Hangzhou China; ^10^ Department of Pathophysiology and Transplantation Università degli Studi di Milano Milan Italy; ^11^ Transfusion Medicine Unit Fondazione IRCCS Ca’ Granda Ospedale Maggiore Policlinico Milan Italy; ^12^ Biological Resource Center Unit Fondazione IRCCS Ca’ Granda Ospedale Maggiore Policlinico Milan Italy

**Keywords:** liver transplantation, macrosteatosis, mechanism, metabonomic, prognosis, transcriptomics

## Abstract

To identify the mechanism underlying macrosteatosis (MaS)‐related graft failure (GF) in liver transplantation (LT) by multi‐omics network analysis. The transcriptome and metabolome were assayed in graft and recipient plasma in discovery (*n* = 68) and validation (*n* = 89) cohorts. Differentially expressed molecules were identified by MaS and GF status. Transcriptional regulatory networks were generated to explore the mechanism for MaS‐related inferior post‐transplant prognosis. The differentially expressed molecules associated with MaS and GF were enriched in ferroptosis and peroxisome‐related pathways. Core features of MaS‐related GF were presented on decreased transferrin and impaired anti‐oxidative capacity dependent upon dysregulation of transcription factors hepatocyte nuclear factor 4A (HNF4A) and hypoxia‐inducible factor 1A (HIF1A). Furthermore, miR‐362‐3p and miR‐299‐5p inhibited transferrin and HIF1A expression, respectively. Lower M2 macrophages but higher memory CD4 T cells were observed in MaS‐related GF cases. These results were validated in clinical specimens and cellular models. Systemic analysis of multi‐omics data depicted a panorama of biological pathways deregulated in MaS‐related GF. Transcriptional regulatory networks centered on transferrin and anti‐oxidant responses were associated with poor MaS graft quality, qualifying as potential targets to improve prognosis of patients after LT.

## Graphical abstract

Transcriptome and metabolome profiling of graft tissues and recipient plasma revealed that MaS‐related GF molecules were enriched in ferroptosis, peroxisome, and Peroxisome proliferator‐activated receptors (PPAR) pathways. L‐glutathione biosynthesis metabolites were depleted and transferrin‐centric network regulated MaS‐related GF. Decreased transferrin impairs anti‐oxidative capacity via HNF4A/HIF1A. Increased circulating aromatic amino acid amplifies MaS‐induced GF risk. MaS‐induced GF stems from impaired anti‐oxidative capacity via comprehensive regulatory networks.

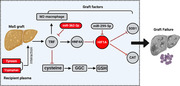

## 1 INTRODUCTION

Liver transplantation (LT) is still the most effective life‐saving option for patients with end‐stage liver disease.^1^ Despite the encouraging progress in xenotransplantation of organs from gene‐edited pigs,^2^ donations from deceased individuals will remain the major source of grafts for LT in the foreseeable future.^3^ However, LT outcomes are affected by quality and function, which are negatively affected by the population aging, diabesity, and fatty liver disease pandemic.^4^ To relieve organ shortage, marginal livers (e.g., with steatosis) are considered for clinical use, but their use can negatively impact post‐transplant prognosis.^5^

Macrosteatosis (MaS) is the accumulation of fat in large lipid droplets in ≥5% of hepatocytes, which affects more than 30% of LT grafts.^6^ MaS is associated with an increased risk of early allograft dysfunction (EAD) and graft loss.^7^ The poorer prognosis of MaS grafts was attributed to a lower tolerance to lipid oxidative stress, ischemia‒reperfusion injury (IRI), hepatocyte necroptosis, and impaired regenerative capacity.^8^ Deranged peroxisome (PER) causes more lipid peroxidation (LPO) and ferroptosis (FPT),^9^ which might aggravate the non‐alcoholic fatty liver disease (NAFLD) and IRI progress.^10^ However, the detailed molecular mechanisms of MaS‐related graft failure (GF) remain largely uncharacterized, given the current absence of comprehensive data from donor‒recipient matched samples.

The clarification of the mechanisms linking graft MaS to detrimental prognosis might therefore help clinicians to optimize the utilization of marginal grafts in LT. This mechanistic study might also reveal novel therapeutic targets that can be exploited, for example, during machine perfusion, as protective strategy to improve the quality of marginal grafts.^10^ Given the complexity of LT process, multi‐omics integrative studies combining transcriptomic and metabolomic data may be instrumental to explore mechanisms and potential targets, thanks to the possibility to cross‐validate whole profiles of molecules through systems biology approaches.^11^ However, to date, results from combined omics have rarely been reported in the same cohort with samples from both donors and recipients. Moreover, the transcriptome profiling in clinical LT studies was more commonly assayed by microarrays, but not more advanced RNA‐sequencing (RNA‐seq).^12^

We therefore hypothesized that an integrative omics study with both donor and recipient factors might identify mechanistic pathways and potential therapeutic targets associated with MaS‐related GF. To this end, we prospectively enrolled a clinical cohort of 68 LT patients to investigate the mechanisms on potential impacts of graft MaS on post‐transplant complications. Comprehensive omics assays including mRNA/microRNA (miR) sequencing and metabolomics assays were performed on graft tissues and recipient plasma obtained before LT. The main aims of the study were to: (1) identify candidate pathways and key molecules by integrative omics data; (2) build a prognostic model based on multi‐omics data; (3) evaluate the interactions between LT‐related phenotypes and regulatory omics modules by advanced weighted gene correlation network analysis (WGCNA) model; and (4) predict the mechanism underlying these associations based on networks between potential transcription factors (TFs), miRs, mRNAs, and metabolites.

## 2 RESULTS

### 2.1 MaS causes inferior LT outcomes

**FIGURE 1:**
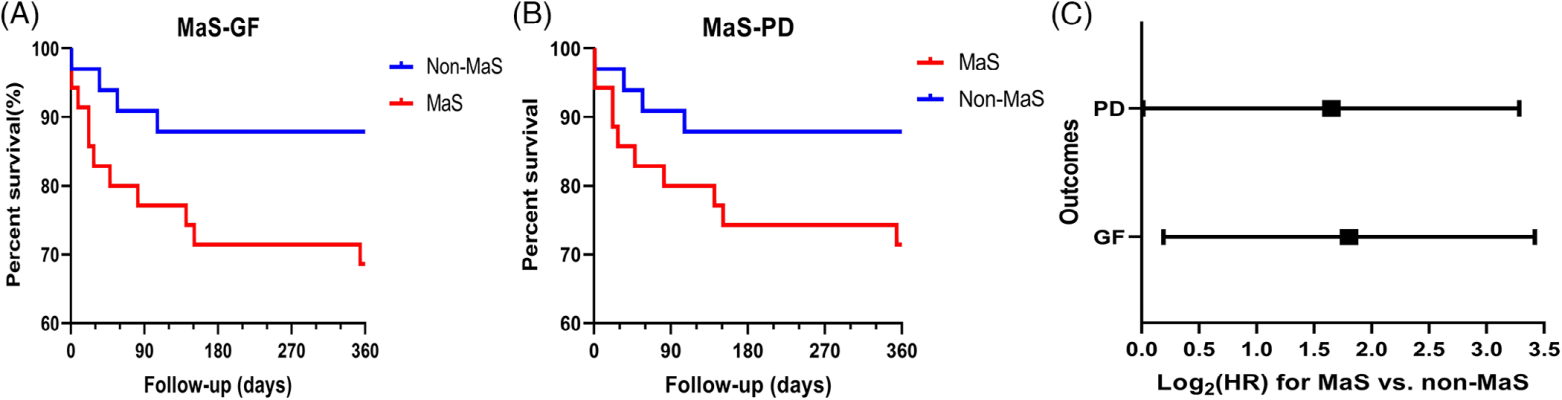
Study flowchart and impact of graft macrosteatosis (MaS) on prognosis after liver transplantation. (A) Time‐dependent curves of graft survival by MaS status. (B) Time‐dependent curves of patient survival by MaS status. (C) Log‐transformed hazard ratios (HRs) of MaS on post‐transplant prognosis. Curves were fitted by Kaplan‒Meier method; survival rates were compared by log‐rank test. HRs were assessed by Cox regression model.

Sixty‐eight patients (accounting for 84% of LT cases during same period at the recruiting hospital) with a median follow‐up duration of 450 days were enrolled in the study. The clinical features of donors, recipients, grafts, surgeries, and their interactions stratified by MaS status are shown in Table 1. More than half of the patients (52.9%) had viral hepatitis. Primary non‐function (PNF) and EAD occurred in three and eight recipients, respectively. Thirty‐five (51.5%) LT cases used grafts with MaS. Increased EAD and elevated alanine aminotransferase (ALT) and aspartate aminotransferase (AST) peak levels were observed in recipients who received MaS grafts (*p* < 0.05, Table 1). MaS presence was associated with inferior post‐transplant prognosis (*p* < 0.05). Hazard ratios (HRs) for MaS on GF and patient death (PD) were 3.49 and 3.14, respectively (*p* < 0.05, Figure 1).

**TABLE 1:** Clinical features of patients who underwent liver transplantation (LT) and were enrolled in the study stratified by macrosteatosis (MaS) status.

Indicators	Non‐MaS	MaS	*p*‐Value
Number	33	35	NA
R‐age (years)	53 (46‒61.5)	55 (48‒60)	0.75
R‐gender (male, %)	26 (78.8)	27 (77.1)	0.87
R‐BMI (kg/m^2^)	22.5 (19.0‒25.1)	22.8 (21.5‒25.0)	0.42
R‐blood type (A/B/O/AB)	9/10/9/5	8/12/10/5	0.97
R‐pre‐operative AFP (ng/mL)	7 (1.7‒16.1)	7 (3.1‒25.9)	0.25
R‐MELD score	24 (16‒40)	27 (16‒40)	0.21
R‐Child‒Pugh score	10 (9‒11)	11 (8‒12)	0.39
R‐HBV infectors (*n*, %)	16 (48.5)	20 (57.1)	0.48
Indications for LT: VH/ALC/PBC/LF/HCC/others (*n*)	12/4/4/8/9/2	17/3/2/16/14/1	0.59
D‐age (years)	48 (32‒56)	46 (32‒50)	0.62
D‐gender (male, %)	27 (81.8)	25 (71.4)	0.31
D‐BMI (kg/m^2^)	22.6 (20.6‒24.5)	24.0 (22.5‒27.2)	0.02
D‐HBV infection (*n*, %)	4 (12.1)	3 (8.6)	0.63
Donation type (DCD/DBD)	17/16	15/20	0.48
D‐serum indicators			
D‐sodium (mmol/L)	145.0 (136.5‒151.0)	156.0 (145.0‒162.5)	<0.01
D‐potassium (mmol/L)	4.3 (3.9‒4.7)	4.1 (3.7‒4.3)	0.04
D‐sodium/potassium ratio	35.0 (30.5‒37.3)	37.7 (34.6‒44.1)	0.01
D‐BUN (mmol/L)	7.9 (4.6‒9.5)	7.6 (4.6‒14.4)	0.44
D‐CR (μmol/L)	77.0 (59.5‒146.8)	113.6 (64.0‒163.0)	0.33
D‐TB (μmol/L)	17.1 (11.2‒30.8)	14.5 (10.5‒22.3)	0.25
D‐ALT (U/L)	51.0 (18.5‒115.0)	42.0 (25.0‒80.0)	0.87
GW/RW × 100	2.1 (1.8‒2.5)	2.3 (1.9‒2.8)	0.27
ABO mismatch (*n*, %)	1 (3.0)	2 (5.7)	0.52
Gender‐mismatched LT (*n*, %)	13 (39.4)	14 (40.0)	0.96
G‐MaS degree (%)	0	8 (5‒15)	NA
G‐fibrosis (none/S1/S2/S3/S4)	16/15/2/0/0	1/26/5/2/1	<0.01
G‐cholestasis presence (%)	7 (21.2)	17 (48.6)	0.02
WIT (min)	6 (1‒11)	6 (1‒13)	0.68
CIT (min)	416 (350‒497)	469 (354‒576)	0.45
Surgical duration (min)	324 (291‒384)	319 (285‒388)	0.67
RBC transfusion (U)	7.0 (3.5‒9.5)	8.0 (3.5‒11.0)	0.66
FFP transfusion (mL)	900 (710‒1100)	905 (750‒1335)	0.43
Intraoperative fluid transfusion (mL)	6180 (4308‒8212)	6270 (4895‒7590)	0.79
Blood loss (mL)	1500 (900‒2350)	1500 (1000‒2800)	0.78
EAD (Y, %)	0 (0)	8 (22.9)	<0.01
PNF (Y, %)	1 (3.0)	2 (5.7)	0.52
ALT peak (U/L)	637 (391‒1332)	1211 (550‒2084)	0.01
AST peak (U/L)	1287 (831‒3155)	2705 (1340‒5946)	0.03
GGT peak (U/L)	293 (178‒521)	245 (155‒384)	0.23
ALP peak (U/L)	297 (182‒374)	256 (163‒400)	0.59
TB peak (μmol/L)	123 (79‒351)	206 (98‒330)	0.27
PT peak (s)	18 (17‒22)	20 (17‒28)	0.13
INR peak	1.6 (1.5‒2.0)	1.8 (1.5‒2.5)	0.13
ICU stay (h)	190 (134‒276)	209 (155‒324)	0.27
LOS after LT (days)	27 (22‒39)	28 (20‒43)	0.86

*Note*: Quantitative data are presented as medians (interquartile ranges) and compared by Mann‒Whitney *U*‐test. Categorical variables are presented as numbers and percentages in the whole cohort and compared by chi‐squared test.

Abbreviations: AFP, alpha‐fetoprotein; ALC, alcoholic liver cirrhosis; ALP, Alkaline Phosphatase; ALT, alanine aminotransferase; AST, aspartate aminotransferase; BMI, body mass index; BUN, blood urea nitrogen; CIT, cold ischemia time; CR, creatinine; EAD, early allograft dysfunction; DBD, Donation after brainstem death; DCD, donation after cardiac death; FFP, fresh frozen plasma; GGT, Gamma‐glutamyl Transferase; GW, graft weight; HBV, hepatitis B virus; HCC, hepatocellular carcinoma; ICU, intensive care unit; INR, international normalized ratio; LF, liver failure; LOS, length of stay; MELD, Model for End‐Stage Liver Disease; PBC,primary biliary cholangitis; PNF, primary non‐function; PT, Prothrombin time; RBC, red blood cell; RW, recipient weight; TB, total bilirubin; VH, viral hepatitis; WIT, warm ischemia time.

### 2.2 Key molecules and pathways from multi‐omics data

**FIGURE 2:**
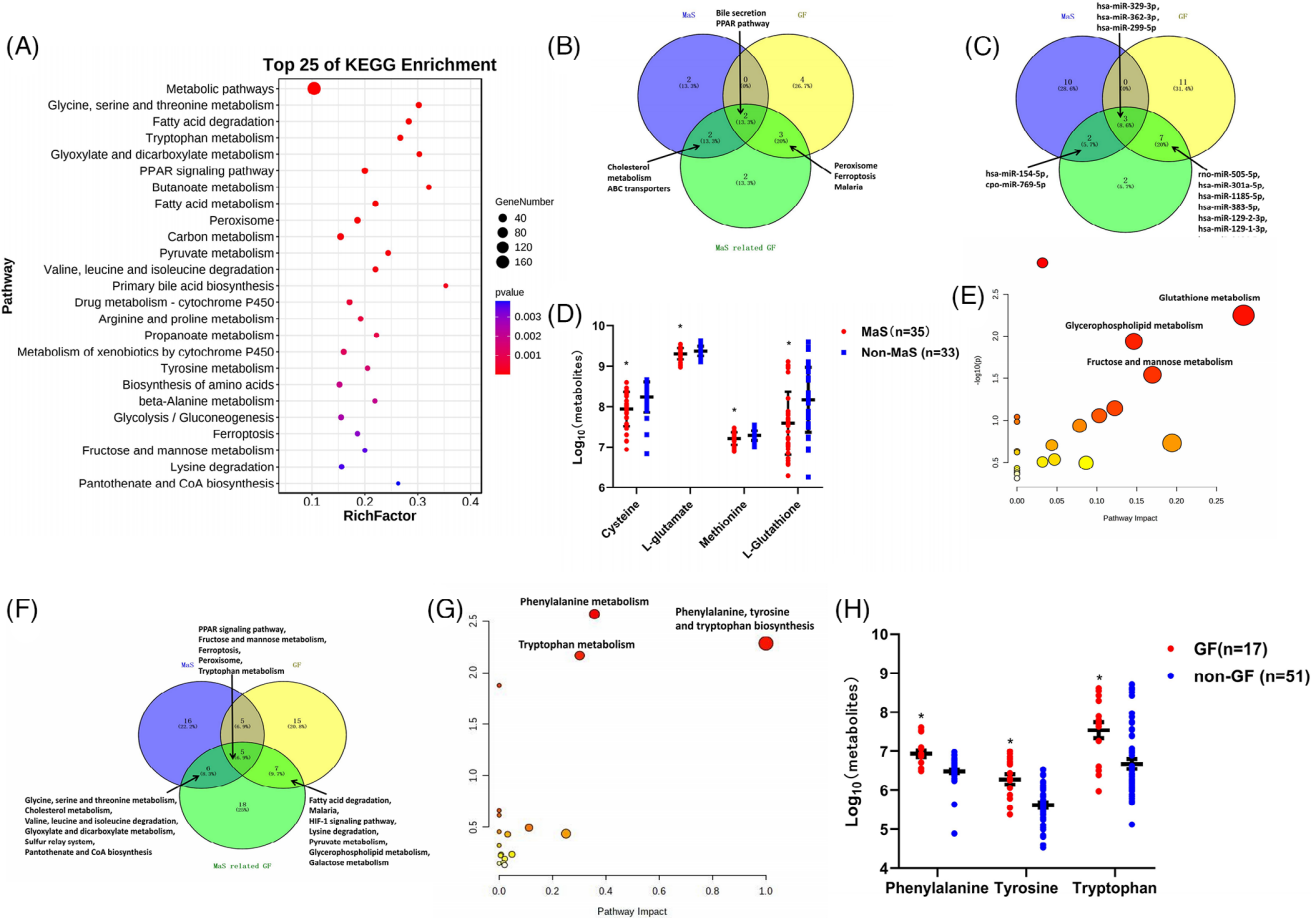
Multi‐omics analysis of samples categorized by macrosteatosis (MaS)‐related graft failure (GF) status. (A) Pathway enrichment based on graft differentially expressed genes (DEGs) associated with MaS‐related GF. (B) Overlapped pathways enriched by graft DEGs for grafts MaS, GF, and MaS‐related GF. (C) Overlapped graft miRNAs associated with grafts MaS, GF, and MaS‐related GF. (D) Variations in key graft metabolites associated with MaS. (E) Pathway enrichment based on down‐regulated graft differentially expressed metabolites (DEMs) associated with MaS‐related GF. (F) Overlapped pathways enriched by integrative graft DEGs and DEMs for grafts MaS, GF, and MaS‐related GF. (G) Pathway enrichment based on up‐regulated recipient plasma DEMs associated with GF. (H) Variations in key metabolites associated with GF in recipient plasma. Blue, yellow, and green circles represent the differentially expressed pathways/molecules in groups with MaS, GF, or MaS‐related GF groups, respectively (panels B, C, F). Asterisk (*) represents statistically significant difference at *p* < 0.05.

rna integrity numbers (RINs) for tissues passing quality control (QC) were 8.7 (interquartile range: 7.7‒9.0, Figure S1). According to the mRNA‐sequencing (mRNA‐seq) data, 1364, 938, and 1430 were identified as differentially expressed genes (DEGs) in grafts with MaS, GF, and MaS‐related GF, respectively (Tables S1‒S3). DEGs associated with MaS, GF, and MaS‐related GF were enriched in 24, 28, and 45 pathways, respectively (*p* < 0.05). The top 25 enriched pathways in MaS, GF, and MaS‐related GF are presented in Figures 2 and S2 and Tables S4‒S6. Most enriched pathways were relevant to energy substance metabolisms (75%, 68%, and 80% MaS, GF, and MaS‐related GF, respectively). Lipid‐related pathways (fatty acid degradation/metabolism) were enriched in samples with GF and MaS‐related GF (Figures 2A and S2B). After excluding metabolic pathways, Venn plot showed that pathways including PPAR signaling, PER, FPT, bile secretion, cholesterol metabolism, ABC transporters, and malaria were enriched and overlapped in both cases with MaS‐related GF and MaS/GF per se (Figure 2B). Dysregulated PER and FPT distributed in Kyoto Encyclopedia of Genes and Genomes (KEGG) “cellular process” classifications were enriched in GF and MaS‐related GF samples.

For miRNA‐seq data, difference was presented on 15, 22, and 14 mature miRNAs in samples with MaS, GF, and MaS‐related GF, respectively (Tables S7‒S9). Three differentially expressed miRNAs (hsa‐miR‐329‐3p, hsa‐miR‐362‐3p, and hsa‐miR‐299‐5p) were deregulated in all groups. Two miRNAs showed significance in MaS and MaS‐related GF groups. Seven miRNAs shared differential expression in GF and MaS‐GF groups (Figure 2C), whereas 170 molecules showed significant variations in MaS grafts (Table S10). Down‐regulated metabolites were mainly enriched in pathways of glutathione, cysteine, methionine, and purine metabolism (Figures S2C and S3 and Table S11). The biosynthetic process from cysteine (C00097) to L‐glutathione (GSH, C00051) was inhibited in MaS grafts. Decrements on key metabolites cysteine (C00097), methionine (C00073), L‐glutamate (C00025), and GSH (C00051) were observed in MaS grafts (Figure 2D). Eight‐eight differentially expressed metabolites (DEMs) were found in cases with MaS‐related GF (Table S12). Down‐regulated DEMs were mainly enriched on pathways including glutathione (GSH) and glycerophospholipid (GP) metabolism (Figures 2E and S4D,E and Table S13). Inactive process from cysteine (C00097) to GSH (C00051) overlapped between MaS and MaS‐related GF.

Differential molecules from transcriptomics and metabolomics data were integrated for pathway imputation. From these, 32, 32, and 36 pathways were found associated with graft MaS, GF, and MaS‐related GF, respectively (*p* < 0.05, Tables S14‒S16). FPT, PER, and PPAR signaling pathways were consistently confirmed to be associated with MaS, GF, and MaS‐related GF (Figure 2F). Fatty acid degradation was also found as enriched pathway relevant to lipid metabolism in cases with GF and MaS‐related GF (Figure 2F). Decrements in GSH biosynthesis and transferrin (TRF) were major features of FPT in cases with MaS‐related GF. All DEGs involved in PER pathway were down‐regulated in patients with MaS‐associated GF.

A total of 1183 molecules for recipient plasma were identified and enrolled for further analysis. A total of 147 DEMs showed deviations in GF group (Table S17). Up‐regulated DEMs were enriched in pathways of aromatic amino acid (AAA) metabolism (Figure 2G). Higher AAA, including phenylalanine, tyrosine, and tryptophan, were observed in GF group (Figures 2H and S3 and Table S18). Circulating phenylalanine and tryptophan were much higher in recipients with Child‒Pugh C cirrhosis (Figure S2D). Graft MaS was associated with worse prognosis by interaction with elevated phenylalanine/tryptophan (positive relative excess risk caused by interaction [RERI] and attributable proportion [AP] index, Table S19).

### 2.3 Correlation networks based on transcriptomics and metabolomics data

**FIGURE 3:**
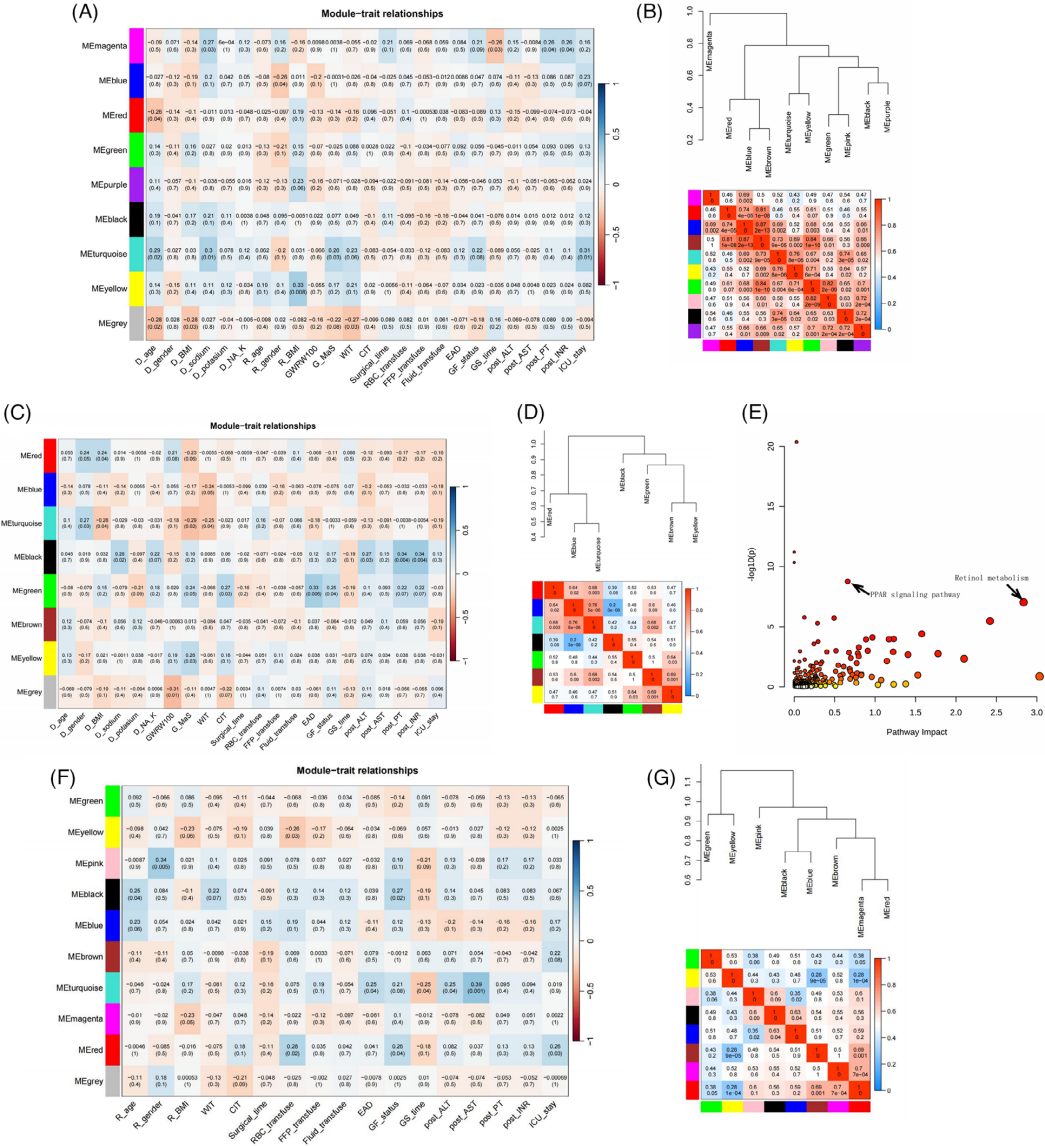
Weighted correlation network analysis based on transcriptome/metabolome and clinical prognostic factors. (A) Relationships between consensus graft transcriptome module and clinical features of liver transplantation (LT) cases. (B) Dendrogram of consensus graft transcriptome module and heatmap of the adjacency obtained by weighted correlation network analysis of the consensus modules. (C) Relationships between consensus graft metabolome module and clinical features of LT cases. (D) Dendrogram of consensus graft metabolome module and heatmap of the adjacency obtained by weighted correlation network analysis. (E) Pathway enrichment based on eigengenes and eigenmetabolites in consensus modules associated with macrosteatosis (MaS)‐related graft failure (GF). (F) Relationships between consensus recipient plasma metabolome module and clinical features of LT cases. (G) Dendrogram of consensus recipient plasma metabolome module and heatmap of the adjacency obtained by weighted correlation network analysis. In panels (A), (C), and (F), each row in the table corresponded to a consensus module, and each column corresponded to a feature. The module name is shown on the left side for each cell. The numbers in the table indicate the correlations of the corresponded module and feature, with the *p*‐values printed below. The table is color coded by correlation according to the color legend. Intensity and direction of correlations are indicated on the right side of the heatmap. In panels (B), (D), and (G), numbers in the table reported the inter‐module correlations, with the *p*‐values printed below. The table was color coded by correlation according to the color legend indicated on the right side of the heatmap.

Eigengenes in turquoise module showed positive associations with donor aging, graft MaS, circulating sodium, intensive care unit (ICU) stay, and GF (Figure 3A). Fifteen pathways were enriched in eigengenes of turquoise module (Figure S4A and Table S20). Pathways, including PER, FPT, and PPAR signaling pathways, were confirmed by eigengenes in turquoise module associated with MaS and GF (Table S20). Metabolites in green module showed close connections with poorer post‐operative GF and EAD, graft MaS, and cold ischemia time (CIT, Figure 3C). Eigenmetabolites in green module were mainly enriched in pathway of histidine metabolism (Figure S4B and Table S21). Connections among graft MaS, CIT, and inferior outcomes were connected by disturbance of histiding metabolism. Eigenmolecules in modules shared by MaS and GF traits were extracted. Nine pathways were enriched when significance was defined at *p* < 1 × 10^−5^ (Figure 3E and Table S22). Retinol metabolism and PPAR signaling pathway were presented significant in joint analysis (Figure S4D,E). Co‐expression networks by recipient plasma metabolomics are presented in Supporting Information.

Post‐transplant GF showed positive associations with eigenmetabolites in black and red modules (Figure 3F). Less connectivity was observed between black and red modules (*p* > 0.05, Figure 3G). Metabolites in black module also showed positive correlation with recipient age. Red module had correlations with higher red blood cell transfusion and longer ICU stay. Further enrichment analysis found that the metabolites in black module were mainly enriched in tryptophan metabolism (Figure S5A,B and Table S23). Recipient aging might cause inferior post‐transplant outcomes via increased tryptophan (C00078, Figure S5B). Molecules in red module were mainly enriched on phenylalanine and GP metabolism (Figure S5C‒E and Table S24). Of note, increased tyrosine (C00082) was key feature for red module. Cause of surgical risks (blood transfusion and delayed recovery) on adverse LT prognosis might be due to their dysregulations on phenylalanine and GP metabolism.

### 2.4 Decreased M2 macrophages infiltration in MaS‐related GF

**FIGURE 4:**
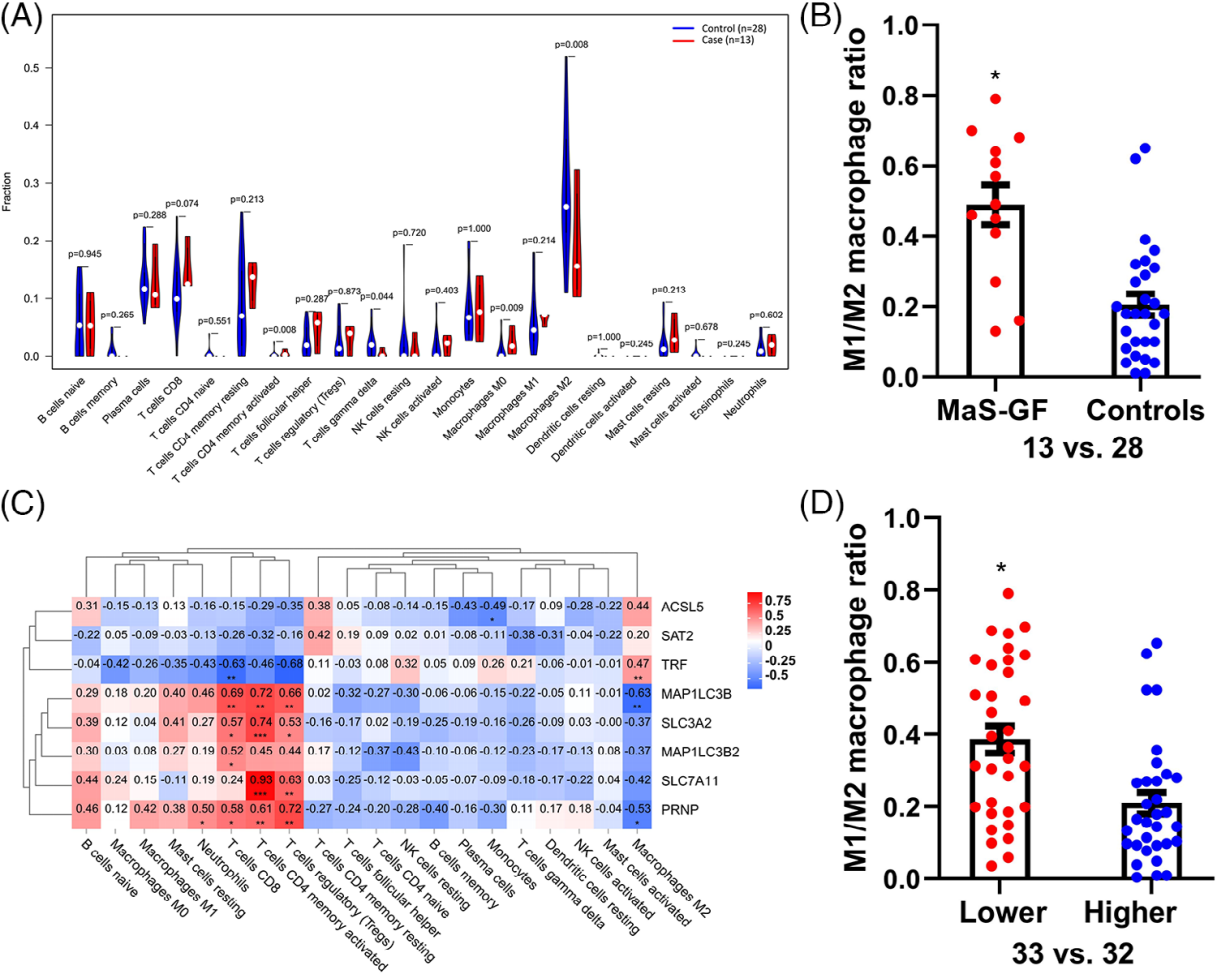
Immune cell infiltration in grafts for liver transplantation. (A) Comparisons of immune cell distributions across cases with macrosteatosis (MaS)‐related graft failure (GF) and controls. (B) M1/M2 macrophage ratio between samples with MaS‐related GF and controls. (C) Correlations between immune cells and genes associated with MaS‐related GF in ferroptosis pathway. (D) M1/M2 macrophage ratio in subjects categorized by transferrin (TRF) expression. Asterisk (*) represents statistically significant difference at *p* < 0.05. Controls were defined for non‐MaS grafts without GF in the follow‐up duration.

Abundances of infiltrated immune cells were evaluated by the CIBERSORT algorithm. Immune cells were divided into 22 fractions and evaluated by percentage. Compared to controls, the fractions of M2 macrophages and gamma‒delta T cells were decreased in cases with MaS‐related GF. By contrast, an increased fraction of activated memory CD4 T cells was observed in MaS‐related GF group (*p* < 0.05, Figure 4A). The ratio of M1 and M2 macrophages was higher in samples with MaS‐related GF (0.49 vs. 0.23, Figure 4B). Correlations between FPT genes and immune cell profiles are presented in Figure 4C. FPT genes could be clustered into two groups. TRF showed significant associations with immune cells in reverse pattern with other susceptive FPT genes. The ratio of M1 and M2 macrophages was higher in patients with lower TRF expression (0.38 vs. 0.19, Figure 4D).

### 2.5 Key regulatory networks in MaS‐related GF process

FPT and PER were proved as key pathways for MaS‐related GF. Inhibited glutathione metabolism and anti‐oxidative activity were observed in FPT and PER pathways. Dysregulated FPT and PER might exert joint effects on MaS‐related GF. Candidate Transcriptional regulatory networks (TRNs) were constructed to reveal details of connection from lipid metabolic disorder to graft dysfunction.

**FIGURE 5:**
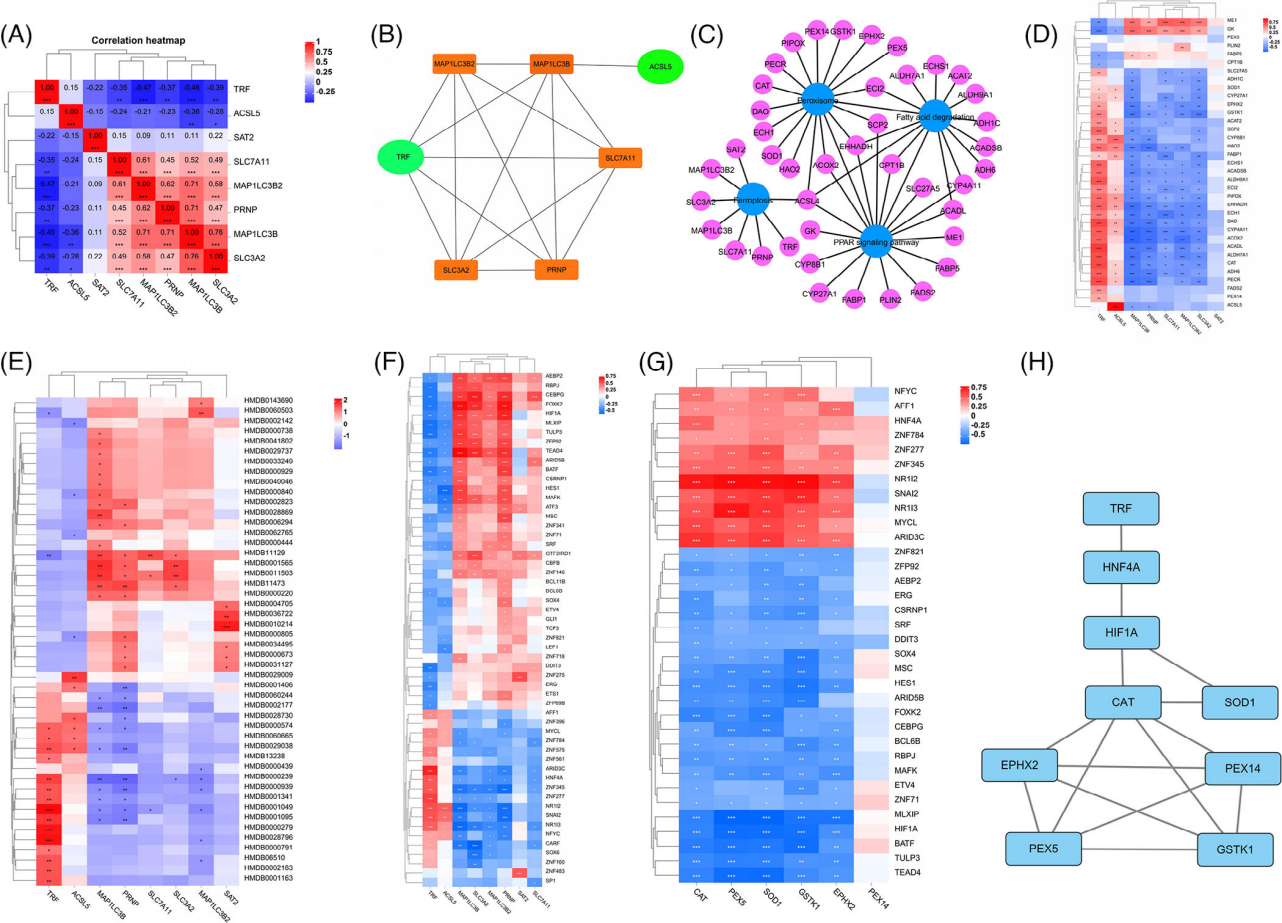
Imputations on regulatory network associated with macrosteatosis (MaS)‐related graft failure (GF). (A) Correlations across ferroptosis genes that associated with MaS‐related GF. (B) Inter‐connections across ferroptosis genes that are associated with MaS‐related GF. (C) Interactions across pathways enriched by positive genes associated with MaS‐related GF. (D) Correlations between ferroptosis genes and lipid‐related genes that are associated with MaS‐related GF. (E) Correlations between ferroptosis genes and metabolomic profiles. (F) Correlations between ferroptosis genes and metabolomic profiles. (G) Correlations between ferroptosis genes and transcription factors profiles associated with MaS‐related GF. (H) Transcriptional regulatory networks between transferrin (TRF) and positive genes in peroxisome pathway based on protein‒protein interaction network (PPIN) analysis. Green circles represent up‐regulation and orange rectangle represents down‐regulation for target genes in cases with MaS‐related GF (panel B), blue circles represent the pathways, and pink circles represent the susceptive genes involved in specific pathways (panel C).

Connections involved in MaS‐related GF can be clustered in correlation heatmap (Figure 5A). TRF was down‐regulated independently in MaS‐related GF samples (Figure 5B). Connections were visualized across positive genes from pathways, including FPT, PER, PPAR signaling, and fatty acid degradation pathways (Figure 5C). Alteration in ACSL4 expression was shared by all four pathways. Genes associated with MaS‐related GF in PER, PPAR signaling, and fatty acid degradation were clustered as lipid‐related genes. TRF showed correlations with anti‐oxidative genes in PER pathway genes, except PEX5 (Figure 5D). With regard to co‐expression between FPT genes and metabolites, TRF showed positive correlations with anti‐oxidative L‐cysteine (HMDB0000574), gamma‐glutamylcysteine (HMDB0001049), and S‐adenosyl‐L‐homocysteine (HMDB0000939) (Figure 5E and Table S25).

Fifty‐five TFs were found to be co‐expressed with FPT genes. Significant fluctuations were observed in 37 TFs followed with TRF increments (Figure 5F and Table S26). Of these, 35 TFs were found to be associated with MaS‐related GF and candidate anti‐oxidative gene expression in PER (Figure 5G and Table S27). catalase (CAT), superoxide dismutase (SOD1), and Glutathione S‐Transferase Kappa 1 (GSTK1) were co‐expressed with all TFs (Figure 5G). Thirteen TFs were co‐expressed with TRF and genes in PER. Network between TRF and reduced anti‐oxidant activity was connected by TFs, including hepatocyte nuclear factor 4A (HNF4A) and hypoxia‐inducible factor 1A (HIF1A). Decreased TRF is linked to reduced HNF4A expression, which may activate HIF1A and suppress CAT/SOD1 (Figure 5H). JASPAR data confirmed the binding sites between HNF4A, HIF1A, and anti‐oxidative CAT/SOD1 with potential binding sites (Figure 5H and Table S28).

Candidate miRNAs with potential impacts on key genes were screened. Six miRNAs (hsa‐miR‐1185‐5p, hsa‐miR‐129‐1‐3p, hsa‐miR‐129‐2‐3p, hsa‐miR‐329‐3p, hsa‐miR‐362‐3p, and hsa‐miR‐154‐5p) were validated to have binding sequence with TRF. Two miRNAs (hsa‐miR‐299‐5p and hsa‐miR‐301a‐5p) had binding sites with HIF1A expression (Table S29). Only hsa‐miR‐362‐3p and hsa‐miR‐299‐5p were co‐expressed with TRF/HIF1A (Table S30). hsa‐miR‐362‐3p may suppress TF expression, and inhibited hsa‐miR‐299‐5p helped to overexpress HIF1A impairing anti‐oxidant activities in MaS‐related GF samples.

### 2.6 Replications of key traits in tissues, cellular model, and validation cohort

**FIGURE 6:**
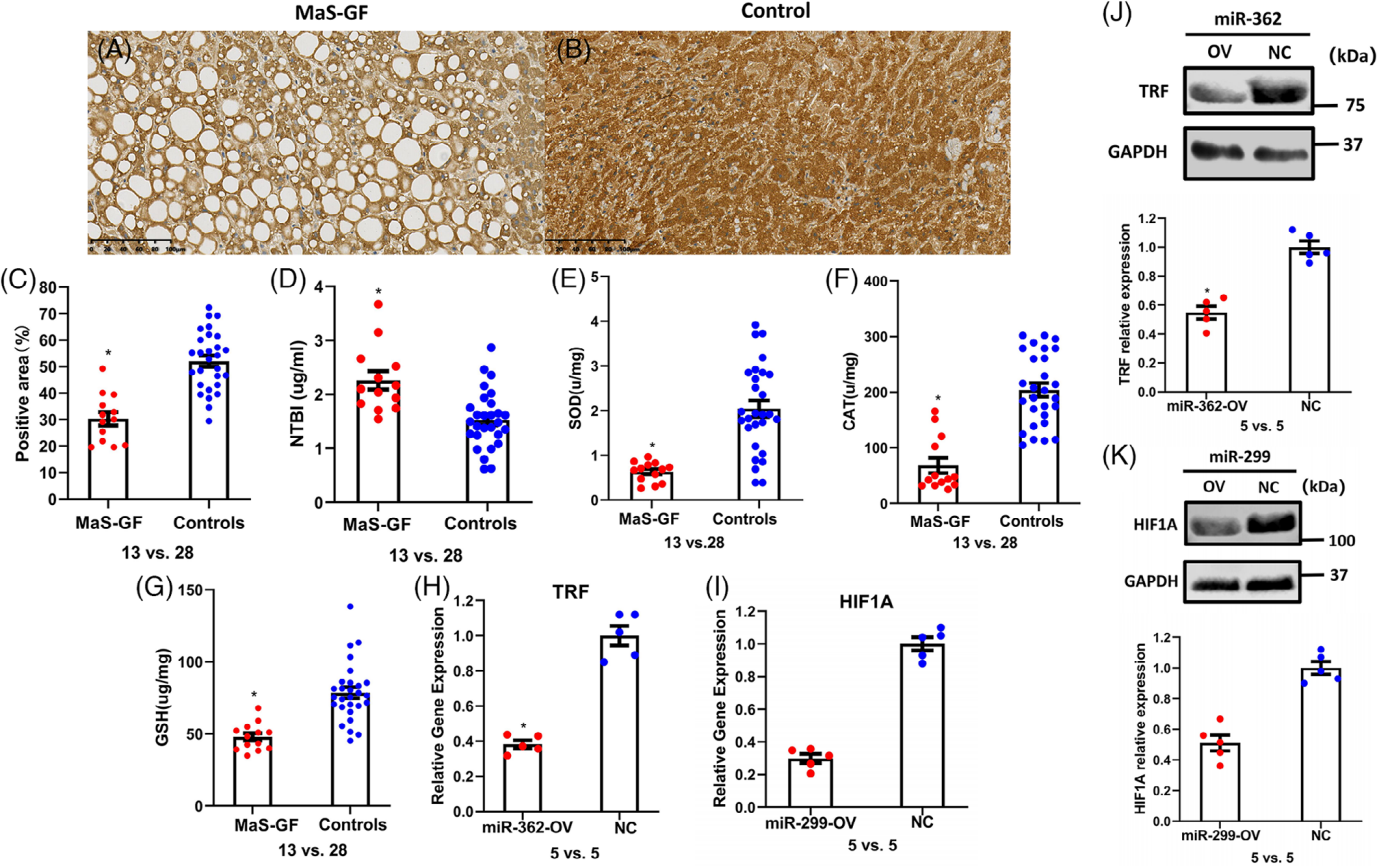
Functional validations of key regulations in graft tissues and hepatocytes. (A) Histological presentation of transferrin (TRF) expression in grafts from cases with macrosteatosis (MaS)‐related graft failure (GF). (B) Histological presentation of TRF expression in grafts from controls. (C) Comparisons of TRF expression in grafts categorized by MaS‐related GF occurrence. (D) Comparisons of NTBI level in donor plasmas categorized by MaS‐related GF occurrence. (E) Comparisons of SOD level in grafts categorized by MaS‐related GF occurrence. (F) Comparisons of CAT level in grafts categorized by MaS‐related GF occurrence. (G) Comparisons of L‐glutathione (GSH) level in grafts categorized by MaS‐related GF occurrence. (H) Comparisons of TRF mRNA in steatotic hepatocytes categorized by miR‐362 overexpression. (I) Comparisons of hypoxia‐inducible factor 1A (HIF1A) mRNA in steatotic hepatocytes categorized by miR‐299 overexpression. (J) TRF protein expression in hepatocytes categorized by miR‐362 expression with blot assay (up) and comparisons (down). (K) HIF1A protein expression in hepatocytes categorized by miR‐299 expression with blot assay (up) and comparisons (down). Original magnification (A and B), ×20; controls were defined as non‐MaS grafts without GF in follow‐up duration; NC was defined as hepatocytes treated with empty vector; asterisk (*) represents statistically significant difference at *p* < 0.05.

Compared to controls without MaS and GF, lower TRF expression was observed in graft tissues with MaS‐related GF occurrence (Figure 6A‒C). Circulatory Non‐transferrin bound iron (NTBI) was highly presented in donor plasma from cases with MaS‐related GF (2.3 vs. 1.5 μg/mL, *p* < 0.05, Figure 6C). For anti‐oxidants, significantly lower SOD, CAT, and GSH were confirmed individually in grafts with MaS‐related failure (Figure 6D‒F). Consistent to omics results, significant lower TRF/HIF1A expression was observed in Oleic acid (OA)‐treated hepatocytes overexpressed by miR‐362 and miR‐299, respectively (Figure 6H,K).

A total of 997 DEGs were found to be associated with MaS‐related GF in validation cohort (Table S31). DEGs were also enriched on FPT, PER, and PPAR signaling pathways (Figure S6). Elevated HIF1A was negatively correlated to other susceptive genes with a trend consistent with that of the discovery cohort (Figure S6B).

## 3 DISCUSSION

Graft MaS is still a major risk factor for graft loss and other post‐transplant complications.^13^ Sharp increments in MaS grafts transplantation were observed following the obesity pandemic and increasing wait‐list times.^14^ The clarification of the molecular mechanisms linking graft MaS to GF is therefore urgently needed to improve graft utilization and develop novel approaches to improve clinical outcomes.

**FIGURE 7:**
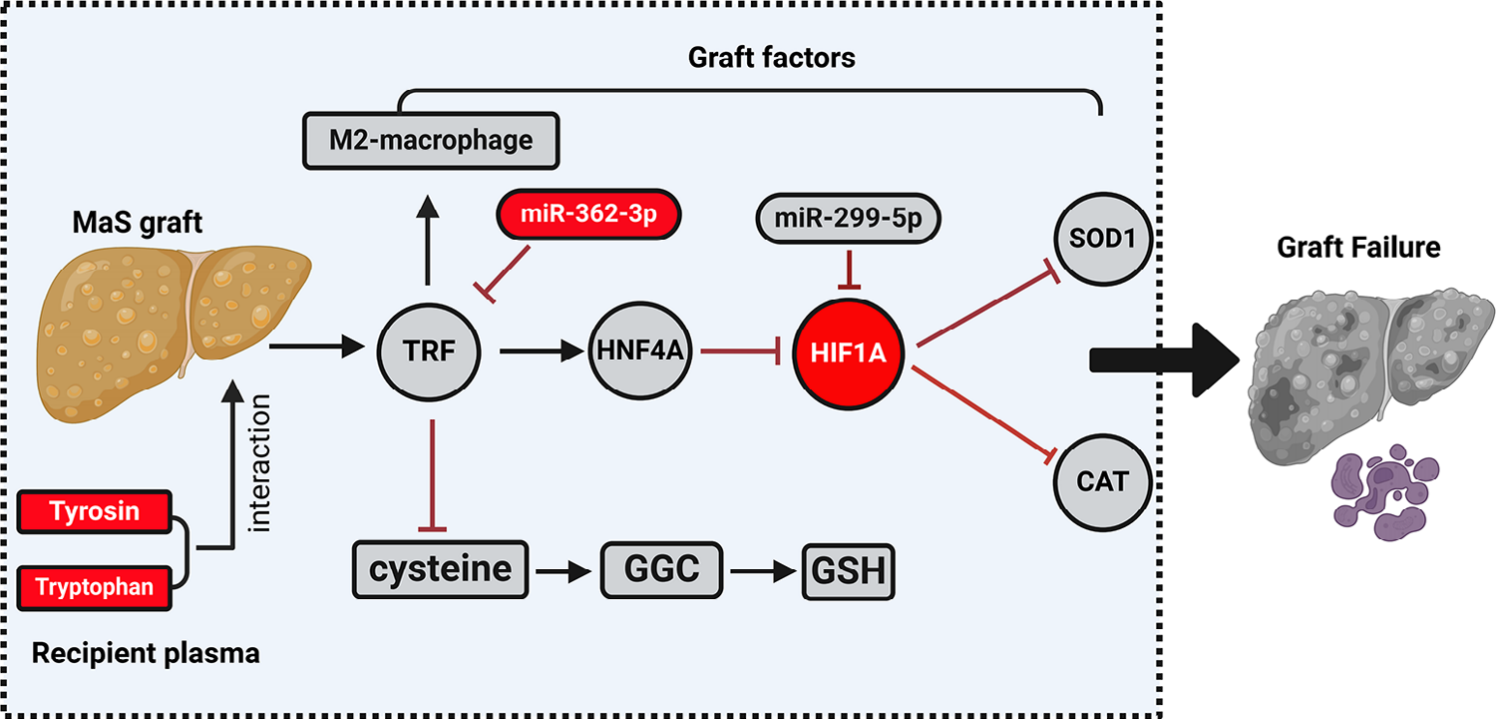
Potential molecular mechanisms for graft failure (GF) caused by macrosteatosis (MaS) based on donor and recipient matched multi‐omics data. Boxes in circular shape represent key genes in process from MaS to GF; boxes in rectangular shape represent key metabolites/specific cell type in process from MaS to GF; boxes in elliptical shape represent key miRNAs in process from MaS to GF; boxes in red represent significant increments; and boxes in gray represent significant decrements. Biorender was applied as tool to generate this figure. GGC, gamma‐glutamylcysteine; GSH, L‐glutathione; HNF4A, hepatocyte nuclear factor 4A; TRF, transferrin.

Here, by exploiting whole profiling of miRNA/mRNA transcriptome and metabolome data from graft tissues and patients’ plasma, we found that: (1) molecules associated with MaS‐related GF were mainly enriched in FPT, PER, and PPAR signaling pathways. (2) Metabolites involved in GSH biosynthesis were exhausted in LT cases with MaS‐related GF. (3) Network centered by TRF is involved in regulating MaS‐related GF. Indeed, down‐regulation of TRF caused impaired anti‐oxidative capacity in MaS grafts via down‐regulation of HNF4A and up‐regulation of HIF1A. (4) miR‐362‐3p and miR‐299‐5p were likely involved in the down‐regulation of TRF and HIF1A. (5) MaS organs with GF had higher M1/M2 macrophages ratio in infiltrated immune cell profiles, which was correlated with oxidative stress. (6) Increased circulating AAA in recipients amplified the risk of MaS on GF. Of note, a validation in an independent local cohort confirmed the importance of FPT in MaS‐induced GF. Overall, results suggested that MaS caused GF via an impaired anti‐oxidative capacity, encompassing decreased TRF expression and FPT through a comprehensive regulatory network (Figure 7).

FPT is a type of programmed cell death regulated by excessive LPO that is dependent of intracellular iron overload.^15^ Lower TRF and GSH are key factors for IRI by FPT,^16^ and were the most distinct features in samples with MaS‐related GF. Low TRF exerted detrimental effects on NAFLD, cirrhosis, and post‐transplant GF by favoring iron overload.^17^ Echoed with these findings, the NTBI, reflecting free circulating toxic iron through oxidative stress, was elevated in MaS grafts donor circulation.^18^ Vice versa, GSH depletion indicated an impaired anti‐oxidative capacity under reactive oxygen species (ROS) in NAFLD patients.^19^ Intriguingly, RNA‐seq data revealed that the MaS grafts attempted to strengthen the glutamate uptake via reverse transport to support GSH synthesis (system Xc constituted by SLC7A11 and SLC3A2). However, this compensatory mechanism could not reverse the GSH depletion and fate of graft loss. A major mechanism for MaS‐related GF could therefore be traced back to the down‐regulation of TRF and glutamate protecting against LPO by GPX4.

PER is an organelle with multi‐functions to maintain the balance between ROS production and scavenging by fatty acid beta‐oxidation and intrinsic anti‐oxidant activities, respectively.^20^ External stresses such as fatty acid overload can break the redox balance in PERs via impairments on anti‐oxidative capacity caused by decreased CAT and SOD1.^9^ In this study, we found that disordered PER function was prominent in samples from MaS‐related GF. Specifically, down‐regulations were found in whole profile of DEGs involved in PER pathways. Further protein‒protein interaction network (PPIN) analysis found that the anti‐oxidative regulatory network was centered on SOD1 and CAT. The peroxisomal anti‐oxidative capacity might be exhausted by ROS related to excess free iron in MaS grafts. Supporting our observations, the down‐regulation of non‐enzymatic anti‐oxidants was also reported in a previous study in this setting.^21^ Otherwise, inhibited FA catabolism and beta‐oxidation was consistent with the mechanism of steatosis in NAFLD.^22^ This result was echoed by pathway enrichment results in fatty acid degradation. The poorer energy metabolism in MaS grafts was also in line with previous results from our group.^23^ Taken together, disturbances in peroxisomal metabolism were linked to MaS‐related GF. Impaired anti‐oxidative capacity in MaS organs possibly induced FPT progression by facilitating cellular death, resulting in graft loss.

Macrophage can be functionally divided into M1 and M2 subtypes under different stimulation.^24^ In our study, disproportionate decrements of M2 macrophage and up‐regulated memory CD4 T lymphocytes were observed in donor livers with MaS‐related GF and lower TRF expression. These results indicated that the disturbed immune cell infiltration in grafts that related to inflammation and rejection might be involved in poor prognosis caused by lipid infiltration. Immune cells might be potential therapeutic targets to improve the graft quality and post‐operative outcomes.

Increased AAA was determined by impaired hepatic metabolic clearance and hepatic blood flow in patients with advanced liver cirrhosis.^25^ In our study, AAA was prominently associated with GF based on whole profiling of metabolome in pre‐operative recipient plasma. Phenylalanine was about fourfold higher in patients with Child‒Pugh C class. AAA exerted synergistic effects on GF by interaction with graft MaS. Steatotic livers should not be allocated to patients with higher AAA to avoid additive risk on inferior prognosis.

By looking at TRNs, variations on transcript factors, including HNF4A and HIF1A, have also been involved in MaS‐related GF. HNF4A plays critical roles in modulating lipid metabolism and metabolic derangement in patients with NAFLD.^26^ Huang et al. found that the targeted activation on HNF4A effectively improve the insulin resistance and NAFLD.^27^ As key element in the adaption to cellular hypoxia, HIF1A was reportedly elevated in patients with NAFLD.^28^ Decreased levels of anti‐oxidant enzymes were also observed in livers under hypoxia status.^29^ Consistently, extremely high HIF1A may indicate the presence of hypoxia in MaS grafts during the LT ischemia phase. Decreased HNF4A in MaS grafts may underpin the reduction in TRF expression, as TRF is regulated by this TF binding to consensus sequences at its promoter. At the same time, HIF1A up‐regulation may also be implicated in altering SOD and CAT expressions. Core TRNs centered by HNF4A and HIF1A might connect the process between FPT and anti‐oxidant impairments in MaS grafts, which was confirmed in in vitro experiments and in the validation cohort.

The miRs could also be involved in mediating the core TRN from MaS to GF. Of which, TRF and HIF1A were confirmed as targets down‐regulated by miR‐362‐3p and miR‐299‐5p via potential binding sites and co‐expression networks. HIF1A may modulate PER pathways during the ischemia‒reperfusion process,^30^ which was also reported as candidate target for miR‐299‐5p.^31^ miR‐362‐3p was reported to activate FPT via binding non‐heme ferritin in hepatoma cells.^32^ Notably, this regulatory network observed in graft omics data could be validated in vitro. To summarize, miR‐299‐5p and miR‐362‐3p could be involved in regulating PER and FPT pathways possibly via HIF1A and TRF, respectively.

A previous study from our group found that graft MaS played vital role in deterioration of post‐transplant prognosis,^33^ which was amplified by interaction with other factors such as limited graft size.^34^ Lipidomic analysis found that the phosphatidylcholine (PC) and phosphatidylethanolamine (PE) decrement in GP metabolism was linked to graft MaS and failure.^23^ Maintenance of PC and PE homeostasis on hepatic cellular membranes are dependent on the availability of reduced GSH linked to LPO, as a key process leading to ferroptosis during ischemia phase.^35^ Indeed, PC oxidation is considered a hallmark of FPT in tissues suffering IRI.^36^ The current findings complement this previous one by providing more detailed molecular mechanisms linking MaS and LPO to disturbances in iron metabolism, transcriptional changes, and cell death leading to GF.

An impaired tolerance of MaS grafts to IRI was contributed to impaired anti‐oxidative capacity caused by inactivation of anti‐oxidants,^37^ suggesting therapeutic interventions to improve the graft quality. The protective effects of anti‐oxidants (such as GSH) on IRI of MaS grafts was confirmed in recent study.^38^ In our study, reduced expression of anti‐oxidative enzymes (GSH, SOD1, and CAT) emerged in core TRN for MaS‐related GF based on multi‐omics data, suggesting that a supplementation of anti‐oxidant complex for marginal grafts during the ischemia phases might help to prevent severer LPO following re‐perfusion after transplantation. Indeed, despite the inferior prognosis, MaS grafts were apparently able to re‐induce GPX4 as key enzyme for maintenance of the FPT process.^39^

The main novelties of the present study can be summarized as follows: (1) this is the first multi‐omics prospective study, where pre‐operative tissue and plasma were collected for comprehensively constructing donor‒recipient matched model. (2) We observed sharp decrements in anti‐oxidant capacity in MaS grafts, highlighting a key role of deranged iron metabolism and lipototoxicity, mutually confirmed by transcriptome and metabolome data. (3) Key networks for MaS‐related GF were depicted by links between miR, TF, target genes, and metabolites, highlighting potential therapeutic targets.

Limitations of our study should be noted as well. First, graft samples were only collected during the ischemia phase and dynamic variations for the same grafts could not be assayed during the IRI process. Second, causal relationship between TRNs and key traits could not be confirmed in experimental models. A well‐designed IRI model in mice with preset sampling time points will be necessary to examine the longitudinal changes and test potential therapeutic targets during the IRI process. Third, individual cellular functions and their interconnections could only be traced through bulk sequencing. Single‐cell RNA‐seq (scRNA‐seq) in LT grafts has provided unique insight into cellular functions and their communication caused by MaS while it was assayed in rats.^40^ Furthermore, scRNA‐seq or spatial RNA‐seq is deserved to clarify the individual function for different cells in human grafts. Forth, omics results were not validated in experimental settings. A well‐designed rat LT study is ongoing for validation. Fifth, the number of participants included in transcriptomics and metabolomics analyses was slightly different due to technical limitations. However, the confounding effect was likely limited because the differentially expressed molecules were assessed independently in individual omics data before final integration. Sixth, heterogeneity was inevitable because of individual differences across clinical samples, although this may also be viewed as a strength because results reflect the clinical practice. Furthermore, a validation cohort was included confirming the main study findings, thereby enhancing the robustness of the study conclusions.

In conclusion, a regulatory network for MaS‐related GF was constructed based on comprehensive integration of multi‐omics data from matched recipients’ sera and graft tissues. Graft MaS was associated with dysregulation of FPT and PER pathways. TRN centered on HNF4A and HIF1A, leading to dysregulations of TRF and anti‐oxidants, was likely involved in modulating the IRI process in MaS grafts. Additional studies are warranted to confirm, extend, and examine the translational relevance of these findings.

## 4 MATERIALS AND METHODS

### 4.1 Study design

**FIGURE 8:**
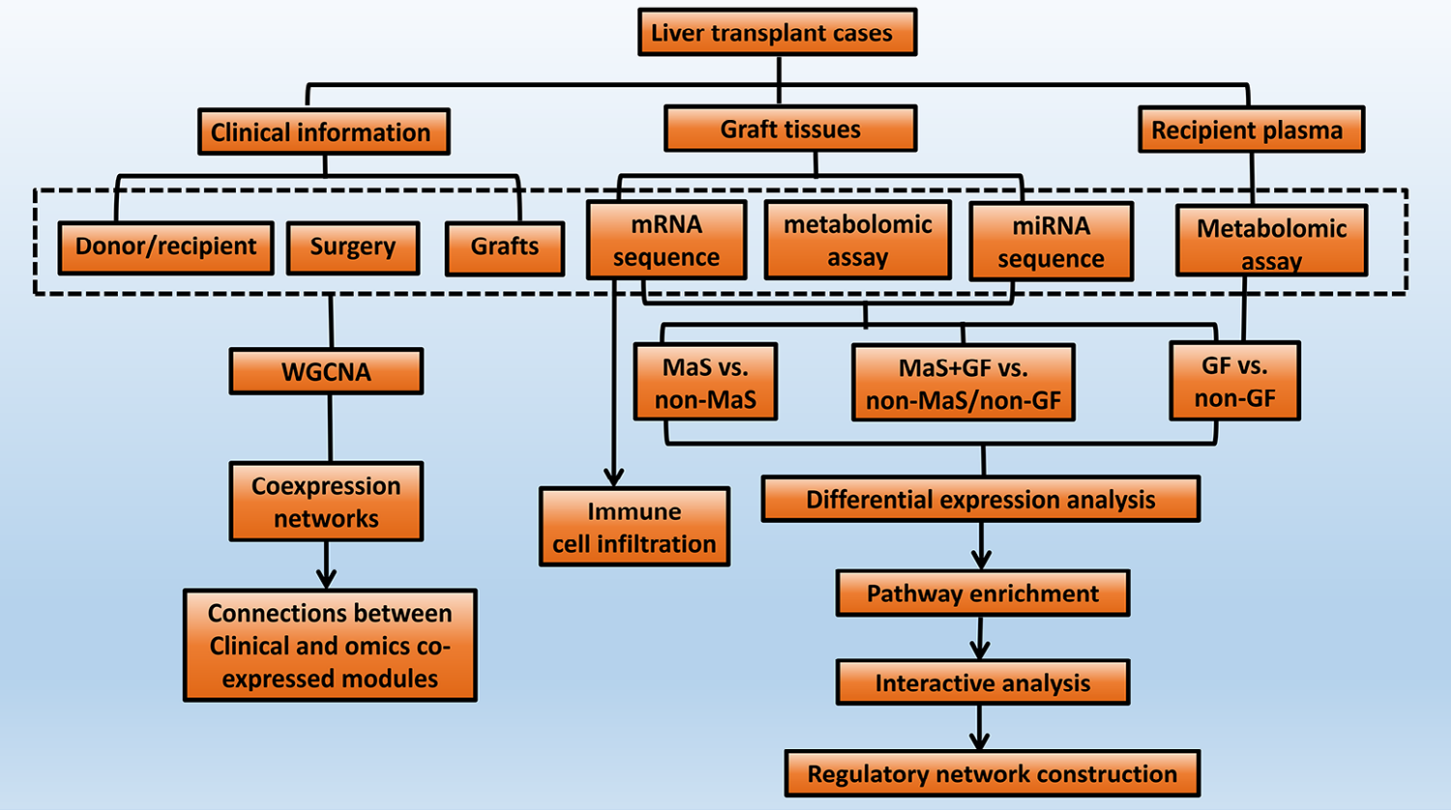
Study flow diagram. GF, graft failure; MaS, macrosteatosis.

The study flow diagram is shown in Figure 8. Graft tissue and recipient plasma of LT cases were collected. RNA‐seq was assayed for miR and mRNA profile in grafts. Liquid chromatography‒mass spectrometry was assayed for metabolite profiles in grafts and recipient plasma. Comparisons were performed in groups categorized by MaS, GF, and MaS‐related GF for DEGs/DEMs clusters. Key pathways were imputed via enrichment of differentially expressed molecules.^41^ Immune cell infiltration was estimated via deconvolution network analysis of bulk transcriptomic data.^42^ Functional modules were clustered by WGCNA.^43^ Regulatory axis for MaS‐related GF was further screened based on correlations and potential binding sites.^44^

### 4.2 Enrollment criteria for qualified LT cases

Deceased donor LT cases were enrolled in Shulan (Hangzhou) Hospital affiliated to Zhejiang Shuren University between December 1, 2020, and May 1, 2021. Patients received LT for end‐stage liver disease. Liver grafts were obtained by local organ procurement organization from donation after citizens’ death. Recipient venous blood was obtained before LT. Inclusion criteria for LT recipients were as follows: (1) adult donor and recipients (≥18 years); (2) non‐multi‐organ transplantation; (3) non‐living donor LT; (4) non‐re‐transplantation; and (5) samples available for acquirement. The study protocol was performed according to the Declaration of Helsinki^45^ and approved by the Zhejiang Shuren University (no. 2020‐093). No graft was acquired from executed prisoners.

### 4.3 Clinical assessment and sample preparations

Clinical variables were collected from medical records. Histological features were assessed based on hematoxylin and eosin slides from biopsied specimens and evaluated by one experienced pathologist by unified scoring systems.^46^ MaS was defined histologically by the presence (in ≥5% of hepatocytes) of large lipid droplets pushing the nucleus to edge of cytoplasm border.^47^ Disease severity before LT was evaluated by the Model for End‐Stage Liver Disease and Child‒Pugh scores.^48^ Recipient hepatocellular carcinoma was determined by pathological examination. EAD and PNF were diagnosed according to established criteria.^49^ Briefly, EAD was diagnosed in recipients meeting the following criteria in first post‐operative week: (1) liver damage (ALT > 3000 IU/mL or AST > 6000 IU/mL); (2) jaundice (total bilirubin ≥ 10 mg/dL); and (3) coagulation dysfunction (international normalized ratio ≥ 1.6). PNF was defined as patient's death or irreversible GF necessitating re‐transplantation in first 3 post‐operative days (PODs). Occurrences of patient death, GF, EAD, or PNF were considered as main adverse LT outcomes. Peak values for indicators of hepatic and coagulation functions in 7 PODs were set as secondary outcomes. Follow‐up information, including survival status/duration or GF cause, was routinely collected via telephone interview every 2 weeks. Donor liver tissues were collected from wedge biopsies of grafts during the early ischemia phase.^23^ Recipient plasma samples were obtained from fasting venous blood sample before LT and stored in ultra‐low temperature freezers until omics investigation.

### 4.4 Transcriptomics and metabolomics assays

Due to RNA degradation, three samples did not pass the quality assessment for transcriptomics assays on mRNA, whereas two samples did not pass QC for miRNA transcriptomics. Therefore, 65 and 66 tissue samples were enrolled for mRNA and miRNA‐seq, respectively. Metabolomic assays were conducted in all 68 graft tissues and recipient plasma samples. Methods for transcriptomics and metabolomics assays are described in Supporting Information.

### 4.5 Validation assays in clinical samples and cellular model

Details for procedure of the validation in graft tissue and cellular model are described in Supporting Information. Information on primer sequences and primary antibodies is provided in Tables S32 and S33.

### 4.6 Validation in independent cohort

An independent validation cohort was enrolled at the same center. LT was performed between December 1, 2021, and August 31, 2022. Graft samples were collected for transcriptomics assays. Enrollment criteria and experimental procedures for omics assays were kept constant. Eighty‐nine LT cases with available graft tissues and complete information were enrolled in the validation cohort. The clinical features for external validation are described in Table S34.

### 4.7 Statistical and bioinformatic assays

Covariates were compared in groups categorized by MaS or GF status. One‐way analysis of variance, Mann‒Whitney *U*‐test, and chi‐squared test were adopted for quantitative and categorical data, respectively. The HR of graft MaS on post‐transplant GF and PD were assessed by Cox regression models. RERI and AP were applied for synergistic effects between graft and recipient factors. The RERI, AP, confidence intervals, and correlated covariance were calculated as described before.^50^ RERI and AP > 0 indicated positive interactions.

Differential expression analyses (DEAs) on graft omics data were conducted in groups categorized by key traits, including graft MaS, GF, and MaS‐related GF status. For omics data from recipients, DEA was only conducted in groups categorized by GF status. Differential analysis was performed by limma algorithm for mRNA/miRNA‐seq data.^51^ Significance of DEGs/miR was determined by *p*‐values estimated by eBayes function. Furthermore, KEGG pathway enrichment analysis based on DEGs was performed and visualized via bubble and Circos plot generated via OmicShare platform (https://www.omicshare.com). Comparisons were performed by Wilcoxon rank sum/*t*‐test for data in normal/non‐normal distribution in metabolomics data. Pathways enrichments on DEMs were imputed by MetaboAnalyst (https://www.metaboanalyst.ca/) with significance defined at *p* < 0.05 and impact >0.1. Integrative pathway analysis was imputed simultaneously based on DEGs and DEMs of interest from transcriptomics and metabolomics data via joint‐pathway model by MetaboAnalyst^52^ and visualized in PATHVIEW (https://pathview.uncc.edu/).

WGCNA was applied to evaluate functional module related to MaS and GF based on global omics data in a scale‐free model.^53^ Correlations between molecule significance and module membership were developed to evaluate the intra‐module connectivity. Molecules from significant modules were extracted for pathway imputation.^54^ Regulatory axes, including miR, mRNA, and TFs for key traits (MaS and GF) were constructed based on co‐expressed modules in enriched pathways. TF list was downloaded online (http://bioinfo.life.hust.edu.cn). PPINs were build based on DEGs and correlated TFs in STRING (https://cn.string‐db.org/).

TRNs were constructed on connections between TFs and targeted genes validated by binding sites in JASPAR.^44^ Connections between miRs and target genes were validated in at least two of three databases (miRDB [http://www.mirdb.org/], miRTarBase [https://mirtarbase.cuhk.edu.cn/], and TargetScan [https://www.targetscan.org/v]). Interactions for enrolled molecules across significant functional pathways were visualized by Cytoscape (v 3.9.0) platform.^55^ Immune cell infiltration was estimated by the CIBERSORT algorithm.^56^ Abundance of 22 immune cell types was profiled based on the expression of characteristic matrix genes from http://cibersort.stanford.edu/ by 1000 permutations. Infiltrated immune cells distributions are listed for each sample. Connections between key genes and pathways (e.g., FPT) and immune cell proportions were presented by correlation heatmaps. Violin plots were mapped to present the different landscape of immune cell profile in cases categorized by MaS and GF.

SPSS (v 26.0, IBM) and R (v 3.5.1; R Foundation) were employed for statistical tests. Analyses on clinical covariates were performed by SPSS. Comparisons on expression or cell infiltration via omics data were conducted by R software. Details on algorithms are summarized in Table S35. Two‐sided *p*‐values <0.05 were considered as statistically significant.

## AUTHOR CONTRIBUTIONS

Zhengtao Liu and Shusen Zheng conceived and designed the study. Lu Yu and Jun Xu performed the experiments and extracted information. Zhengtao Liu, Hai Zhu, Junsheng Zhao, and Shuping Que analyzed the data. Luca Valenti provided consultation on the data analysis and presentation. Zhengtao Liu and Luca Valenti wrote the manuscript. Lei Geng, Lin Zhou, Luca Valenti, and Shusen Zheng reviewed the manuscript. Zhengtao Liu and Shusen Zheng provided funding support. All of the authors approved the final version of the manuscript for submission. All authors have approved to publish this paper.

## CONFLICT OF INTEREST STATEMENT

The authors declare that the research was conducted in the absence of any commercial or financial relationships that could be construed as potential conflicts of interest.

## ETHICS STATEMENT

The study protocol was performed according to the Declaration of Helsinki and approved by the Research Ethics Committee of Shulan International Medical Colleague, Zhejiang Shuren University (2020‐093). Written informed consent was obtained from all participants.

## Supporting information

Supporting Information

Supporting Information

## Data Availability

The data used to generate the figures are available from the corresponding author upon reasonable request.
