# Supplementary material for: Multi‐omics analysis reveals a crosstalk between ferroptosis and peroxisomes on steatotic graft failure after liver transplantation
Source: MedComm (2020). 2024 Jun 12;5(6):e588. doi: 10.1002/mco2.588 (PMC11167151; doi:10.1002/mco2.588)
Supplement: Supplementary file 1 — Supporting Information [file MCO2-5-e588-s002.docx]

Multi-Omics analysis reveals a crosstalk between ferroptosis and peroxisomes on steatotic-graft failure after liver transplantation

Running Title: Omics on MaS Graft for Liver Transplantation

Zhengtao Liu^1,2,3,4,5*#^, Hai Zhu^3,4,6#^, Junsheng Zhao^1,2#^, Lu Yu^1,5,7^, Shuping Que^8^, Jun Xu^9^, Lei Geng^9^, Lin Zhou^3,4,9^, Luca Valenti^10,11,12*^, Shusen Zheng^1,2,3,4,5,9*^

**Affiliation:**

1. Shulan International Medical College, Zhejiang Shuren University, Hangzhou, 310015, Zhejiang, China.
2. Key Laboratory of Artificial Organs and Computational Medicine in Zhejiang Province, Shulan International Medical College, Zhejiang Shuren University, 310015, Hangzhou, Zhejiang, China.

3. NHC Key Laboratory of Combined Multi-organ Transplantation, Key Laboratory of the diagnosis and treatment of organ Transplantation, CAMS, First Affiliated Hospital, School of Medicine, Zhejiang University, 310003, Hangzhou, China

4. Key Laboratory of Organ Transplantation, Zhejiang Province, First Affiliated Hospital, School of Medicine, Zhejiang University, 310003, Hangzhou, China

5. Shulan Hospital (Hangzhou), 310 002 Hangzhou, China

6. Department of Hepatobiliary Surgery, First Affiliated Hospital of Guangxi Medical University, 530021, Nanning, China

7. School of Medicine, Zhejiang Chinese Medical University, 310053, Hangzhou, China

8. DingXiang Clinics, 310 063, Hangzhou, China

9. Division of Hepatobiliary and Pancreatic Surgery, Department of Surgery, First Affiliated Hospital, School of Medicine, Zhejiang University, Hangzhou, 310003, China.

10. Department of Pathophysiology and Transplantation, Università degli Studi di Milano, 20122, Milan, Italy

11. Transfusion Medicine Unit, Fondazione IRCCS Ca’ Granda Ospedale Maggiore Policlinico, 20122, Milan, Italy

12. Biological Resource Center Unit, Fondazione IRCCS Ca' Granda Ospedale Maggiore Policlinico, 20122, Milan, Italy.

Corresponding author:

*Zhengtao Liu, Shulan International Medical College, Zhejiang Shuren University, Hangzhou, 310015, Zhejiang, China.

Email: [liuzhengtao@zjsru.edu.cn](mailto:liuzhengtao@zjsru.edu.cn)

Luca Valenti, MD, Department of Pathophysiology and Transplantation, Università degli Studi di Milano, Milan, Italy. Precision Medicine Lab, Biological Resource Center Unit, Department of Transfusion Medicine, Fondazione IRCCS Ca’ Granda Ospedale Maggiore Policlinico, via Francesco Sforza 35, 20122, Milan, Italy.

Email: [luca.valenti@unimi.it](mailto:luca.valenti@unimi.it)

Shusen Zheng, Division of Hepatobiliary and Pancreatic Surgery, Department of Surgery, First Affiliated Hospital, School of Medicine, Zhejiang University, Hangzhou, 310003, China.

Email: [shusenzheng@zju.edu.cn](mailto:shusenzheng@zju.edu.cn)

^#^ These authors contributed to this work equally.

**Key Words:** Macrosteatosis; Transcriptomics; Metabonomic; Mechanism; Prognosis; Liver transplantation.

**Supplementary documents**

**Material and method**

**1. RNA sequencing for graft tissue**

Profiling of messenger RNA (mRNA) and microRNA (miRNA) in graft tissues were assayed via respective sequencing. Total RNA was isolated and purified using TRIzol reagent (Invitrogen, USA). The transformed cDNA library was assayed by 2X150bp paired-end sequencing (PE150) on Illumina Novaseq™ 6000 platform (LC-Bio, China) following the vendor's protocol. Comprehensive transcriptomes for all grafts samples were mapped by HISAT2 and merged by gffcompare software ^1^. The miRNA libraries of graft tissues were generated using TruSeq Small RNA Sample Prep Kits (Illumina, USA) and profiled by 1X50bp single-end sequencing on Illumina Hiseq 2500 platform. Unique sequences with length from 18 to 26 nucleotide were mapped to species specific precursors in miRBase database (v22.0) to identify known and novel 3p or 5p derived miRNAs. Quality controls of RNA samples were assayed referred to RNA integrity number (RIN) measured by Agilent 2100 bioanalyzer ^2^. The samples with RIN >7.0 was regarded to be qualified for further experiment.

Metabolome of graft tissue and recipient plasma was assayed by high-resolution tandem mass spectrometer (Q-Exactive, Thermo Fisher Scientific, Germany) based on manufacturer’s instruction. Metabolites were annotated using Compound Discoverer software (v3.3, Thermo Scientific, Carlsbad, USA) by matching the exact molecular mass data (m/z ratio) that recorded in online KEGG and HMDB database. More details for omics assays are presented in the supplementary material.

**1.1 Sample preparation and libraries construction for graft tissues**

A total amount of 1 µg RNA for each sample was used as input material for the RNA sample preparations. Sequencing libraries for mRNA were generated using NEBNext® UltraTM RNA Library Prep Kit (Illumina, San Diego, USA) in accordance with manufacturer’s recommendations. Correspondingly, library of miRNA was prepared according to protocol of TruSeq Small RNA Sample Prep Kits (Illumina, San Diego, USA).

**1.2 Clustering and sequencing**

For mRNA sequencing, the clustering of the index-coded samples was performed on a cBot Cluster Generation System using TruSeq PE Cluster Kit v3-cBot-HS (Illumina, USA) in accordance with manufacturer’s instructions. After cluster generation, the library preparations were sequenced on an Illumina Novaseq platform at 150 bp for paired-end reads. Libraries of miRNA were assayed by sequencing at 50bp at single-end read on Illumina Hiseq2500 platform following the vendor’s recommended protocol.

**1.3 Reads mapping and quantification of gene expression level**

For mRNA, the index of reference genome was built using Hisat2 v2.0.5 and paired-end clean reads were aligned to the reference genome using Hisat2 v2.0.5. The count the reads numbers were mapped to each gene by feature counts v1.5.0. And FPKM for each gene was calculated based on its length and reads count mapped to this gene.

For miRNA, the unique sequences with length between 18 and 26 nucleotide were mapped to specific species precursors in miRBase 22.0 by BLAST search to identify known miRNAs and novel 3p- and 5p- derived miRNAs. Length variation at both 3’ and 5’ ends and one mismatch inside of the sequence were allowed in the alignment. The unique sequences mapping to human specific mature miRNAs in hairpin arms were identified as known miRNAs. The unique sequences mapping to the other arm of human specific precursor hairpin opposite to the annotated mature miRNA-containing arm were considered to be novel 5p- or 3p- derived miRNA candidates. The remaining sequences were mapped to other species precursors (excluding human) in miRBase 22.0 by BLAST search. Mapped pre-miRNAs were further screened by BLAST against the human genomes to determine their genomic locations. The unmapped sequences were further screened by BLAST against the other specific genomes. All obtained miRNAs were used to predict the secondary structures using RNAfold software (http://rna.tbi.univie.ac.at/cgi-bin/RNAWebSuite/RNAfold.cgi).

**2. Metabolomic assay for graft tissue and recipient plasma**

All metabolites in graft tissues and recipient plasma across various periods were assayed by untargeted metabolomics via LC-MS/MS technology using VanquishTM UHPLC system (Thermo Fisher) coupled with an Orbitrap Q Exactive series mass spectrometer (Thermo Fisher).

**2.1 Metabolites Extraction**

100mg liver tissues were grounded with liquid nitrogen. 100μl recipient plasma was thawed on ice and vortexed. Homogenates of samples were re-suspended with pre-cooled methanol (80%) and formic acid (0.1%) by vortex. Then samples were incubated on ice for 5 min and then centrifuged at 15000 rpm/4°C for 5 min.

Supernatant was diluted to final concentration which contained 53% methanol by LC-MS grade water. And samples were then transferred to a fresh tube and centrifuged at 15000 g/4°C for 10 min. Finally, the supernatant was injected into the LC-MS/MS system for analysis.

**2.2 Non-target metabolomic assay and Database search**

Liquid Chromatography with tandem mass spectrometry (LC-MS/MS) analyses were performed using a Vanquish UHPLC system (Thermo Fisher) coupled with an Orbitrap Q Exactive series mass spectrometer (Thermo Fisher) in accordance with manufacturer’s instructions.

Raw data files generated by UHPLC-MS/MS were processed using the Compound Discoverer 3.1 (CD3.1, Thermo Fisher) to perform peak alignment, peak picking, and quantitation for each metabolite. The main parameters were set in accordance with manufacturer’s instructions.

1. **Validation assays in clinical samples and cellular model**

Biopsied liver samples were routinely collected and treated by 10% neutral formaldehyde. Graft tissues embedded in paraffin the slides were stained with hematoxylin-eosin (H&E) for assessment of histological presentation. Transferrin expression was stained by immuno-histochemistry (IHC) staining of specific antibody (17435-1-AP, Proteintech, China) according to manufacturer’s instructions. Quantification on positive transferrin expression was determined by Image-Pro Plus software (IPP.6.0, Silver Spring, USA). Activities of antioxidants were quantitatively evaluated based on colorimetry in graft tissues by commercial kits according to manufacturer's instructions. (S0103/S0057S, Beyotime for Superoxide Dismutase [SOD] and glutathione [GSH]; BC0205, Solarbio for catalase [CAT]). Non-transferrin-bound iron (NTBI) was measured in pre-transplant donor plasma by enzyme-linked immunosorbent assay (ELISA) according to instruction provided by commercial kit (SL3533Hu, Sunlong Biotech, China).

Regulation of candidate miRNAs on target genes was examined in steatotic cellular model. Specifically, HepG2 cells purchased from Meisen Chinese Tissue Culture Collections (CTCC, Zhejiang, China) were incubated in 500 uM oleic acid (S4707, selleck) medium for 24 hours. Then, steatotic cells were transfected by Human MicroRNA Expression Plasmid kits (OriGene Technologies) targeting on miR-362-3p and miR-299-5p for 48 hours, respectively. Transcript/protein expression of target genes (TRF/HIF-1A) in transfected cells were examined by quantitative PCR (qPCR) and western blot (WB) described in previous study ^3^.

**4. Cell culture and transfection**

HepG2 cells purchased from Meisen Chinese Tissue Culture Collections (Zhejiang, China) were selected for validation. Cells were cultured in a humid incubator with 5% CO2 at 37℃. Cells were cultured in RPMI-1640 medium (01-100-1A, BI, Israel) with 10% fetal bovine serum (FBS, 04-001-1ACS,BI,Israel) for 24 hours. Then, cellular transfections were performed for overexpression of miR-362-3p or miR-299-5p by commercial plasmid kit (SC400360/SC400308, Origene, China) in accordance with manufacturer’s instruction. Cells treated by empty vector (pCMV6 mammalian vector, PS100019) were assigned as negative controls (NCs). Total RNA and protein of cells were harvested for further detection in 48 hours after transfection.

For mRNA, GAPDH was assigned as internal reference. The primer sequences and primary antibodies were listed in **Table S1 and S2**. For microRNAs, the RNU6-50P snRNA was assigned as internal reference. Reverse transcription and quantitative PCR (qPCR) was performed by miRNA 1st Strand cDNA Synthesis Kit and miRNA Universal SYBR qPCR Master Mix (MR101/MQ101) respectively according to manufacturer’s instruction. The sequences for primer and stem-loop were listed in **Table S1**. Efficiency of transfection was evaluated based on elevated microRNA expression. Effective transfection was defined as higher than 3 folds for overexpression on target microRNAs compared to NC samples.

**References**

1. Pertea M, Kim D, Pertea GM, Leek JT, Salzberg SL. Transcript-level expression analysis of RNA-seq experiments with HISAT, StringTie and Ballgown. *Nature protocols*. 2016;11(9):1650-1667.

2. Mueller O, Lightfoot S, Schroeder A. RNA integrity number (RIN)–standardization of RNA quality control. *Agilent application note, publication*. 2004;1:1-8.

3. Liu Z, Chen T, Lu X, Xie H, Zhou L, Zheng S. Overexpression of variant PNPLA3 gene at I148M position causes malignant transformation of hepatocytes via IL-6-JAK2/STAT3 pathway in low dose free fatty acid exposure: a laboratory investigation in vitro and in vivo. *American Journal of Translational Research*. 2016;8(3):1319.

**Supplementary figure legend:**

Figure S1 RIN values across samples for RNA-sequence.

Figure S2 Multi-omics analysis on samples by MaS and GF status.

1. Pathway enrichment based on graft DEGs associated with graft MaS.
2. Pathway enrichment based on graft DEGs associated with GF status.
3. Pathway enrichment based on down-regulated graft DEMs associated with graft MaS.
4. Variations of aromatic amino acid by Child-pugh classfication.

* represents statistically significant difference compared to remaining groups at P<0.05.

Figure S3 Pathways enriched based on differentially expressed molecules from omics data.

1. Pathway for cysteine and methionine metabolism enriched by decreased DEMs in MaS grafts.
2. Pathway for glutathion metabolism enriched by decreased DEMs in MaS grafts.
3. Pathway for purine metabolism enriched by decreased DEMs in MaS grafts.
4. Pathway for glutathion metabolism enriched by decreased DEMs in cases with MaS related GF.
5. Pathway for glycerophospholipid metabolism enriched by decreased DEMs in cases with MaS related GF.
6. Pathway for PPAR signaling enriched by DEGs associated with MaS related GF.
7. Pathway for ferroptosis enriched by DEGs/DEMs associated with MaS related GF.
8. Pathway for peroxisome enriched by DEGs associated with MaS related GF.
9. Pathway for fatty acid degradation enriched by DEGs associated with MaS related GF.
10. Pathway for phenylalanine metabolism enriched by elevated DEMs in plasma of recipients with final GF.
11. Pathway for phenylalanine, tyrosine and tryptophan biosynthesis enriched by elevated DEMs in plasma of recipients with final GF.
12. Pathway for tryptophan metabolism enriched by elevated DEMs in plasma of recipients with final GF.

For panel G, yellow circles represent significant elevations of candidate metabolites in case groups; blue circles represent significant decrements of candidate metabolites in case groups; red boxes represent significant elevations of candidate genes in case group; green boxes represent significant decrements of candidate genes in case group. For other panels, red boxes represent significant elevations of metabolites in case groups; green boxes represent significant decrements of metabolites in case groups.

Figure S4 Pathways enriched by eigenmolecules from co-expressed modules in graft omics data

1. Pathway enrichment based on eigengenes from turquoise module.
2. Pathway enrichment based on eigenmetabolites from green module.
3. Pathway for histiding metabolism enriched by eigenmetabolites from green module.
4. Pathway for PPAR signaling enriched by eigengenes and eigenmetabolites from co-expressed module associated with GF.
5. Pathway for retinol metabolism enriched by eigengenes and eigenmetabolites from co-expressed module associated with GF.

For panel C, red boxes represent significant elevations of metabolites in case groups. For panel D and E, yellow circles represent significant elevations of candidate metabolites in case groups; red boxes represent significant elevations of candidate genes in case group.

Figure S5 Pathways enriched by eigenmetabolites from co-expressed modules in metabolomics data of recipient plasma.

1. Pathway enrichment by eigenmetabolites from black co-expressed module.
2. Pathway for tryptophan metabolism enriched by eigenmetabolites from black module.
3. Pathways enrichment by eigenmetabolites from red co-expressed module.
4. Pathways for phenylalanine metabolism enriched by eigenmetabolites from red module.
5. Pathways for glycerophospholipid metabolism enriched by eigenmetabolites from red module.

For panel B, D and E, red boxes represent significant elevations of metabolites in case groups

Figure S6 Validation of key molecules in external cohort.

1. Pathway enrichment based on graft DEGs associated with MaS-related GF.
2. Correlations across genes from key regulatory network.
3. Ferroptosis pathway enriched by DEGs associated with MaS-related GF.
4. PPAR signalling pathway enriched by DEGs associated with MaS-related GF.
5. Peroxisome pathway enriched by DEGs associated with MaS-related GF.

*,**,*** represent statistically significant correlations at P<0.05,0.01 and 0.001 (panel C); boxes in red boders represent DEGs associated with MaS related GF in validation cohort (panel C, D, E)

**Figure S1**


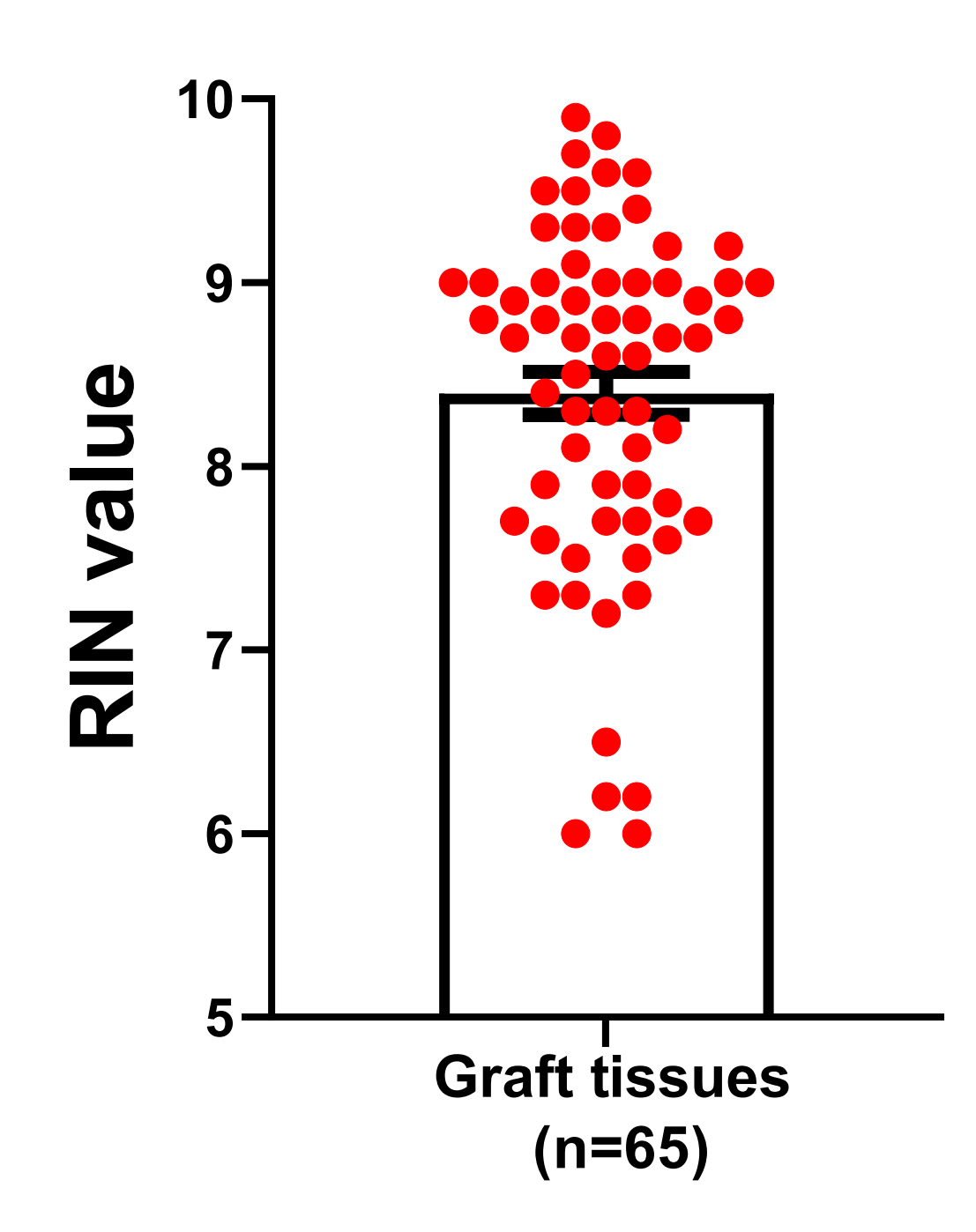


**Figure S2**

**
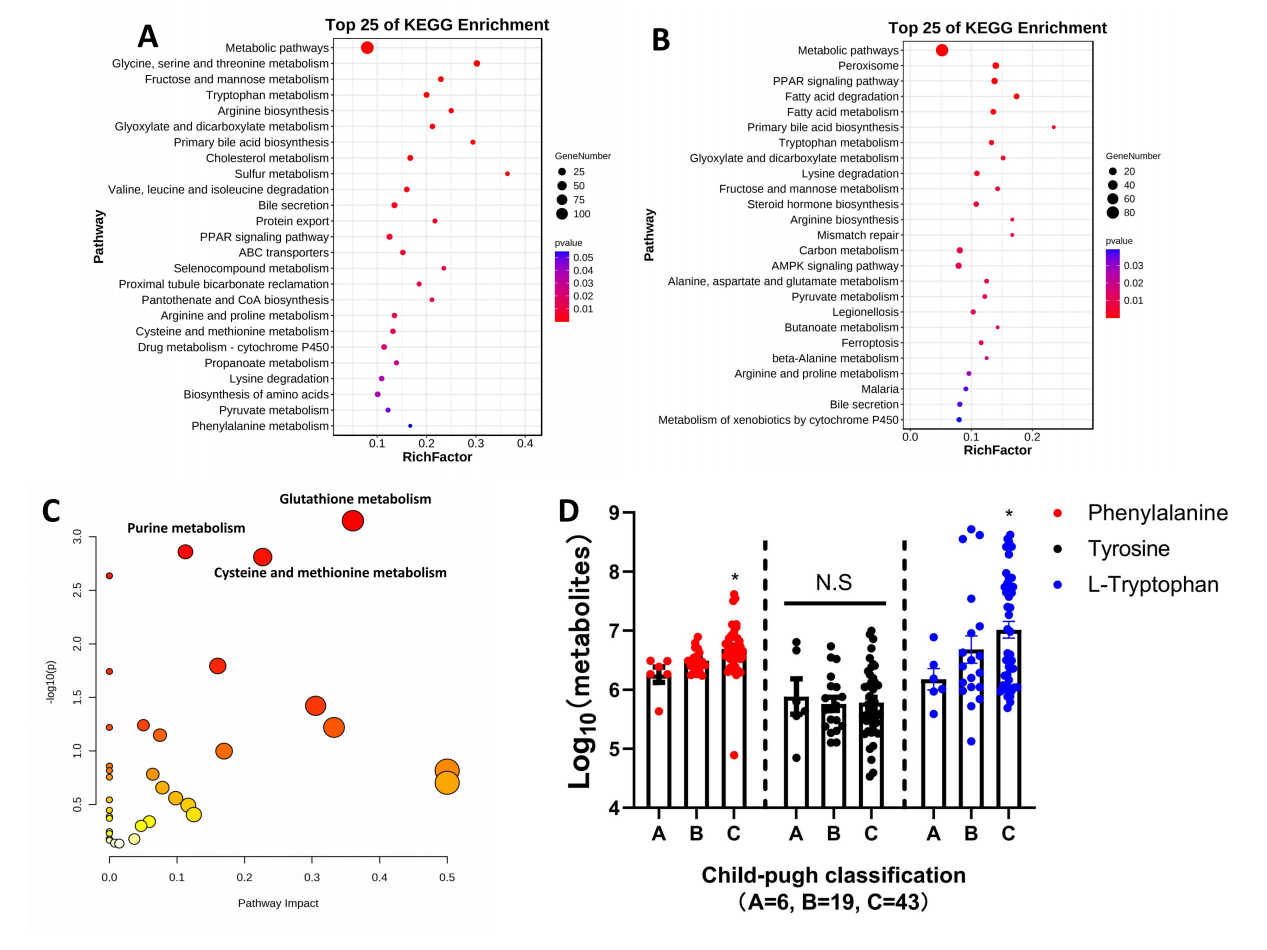
**

**Figure S3**

**
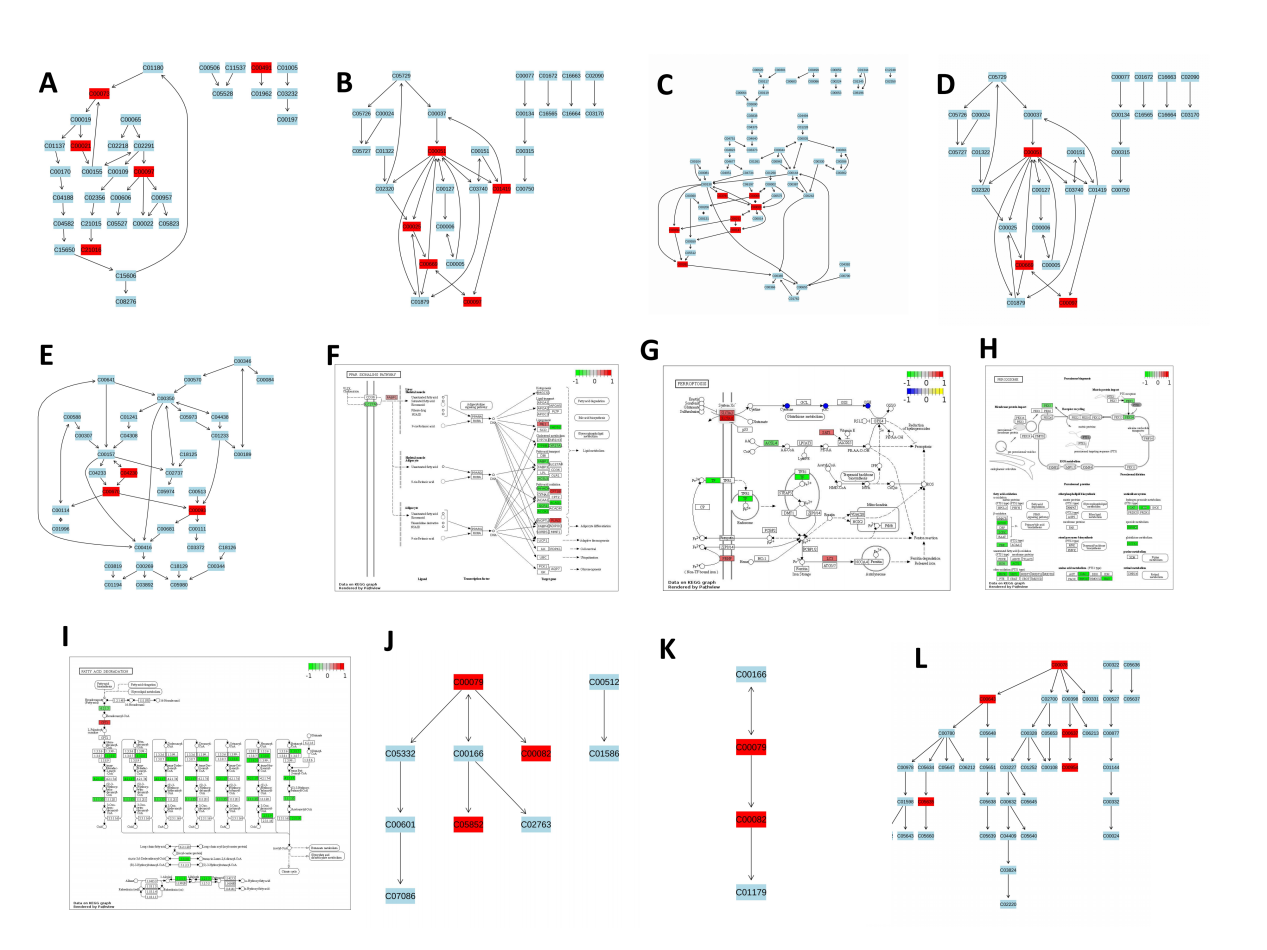
**

**Figure S4**

**
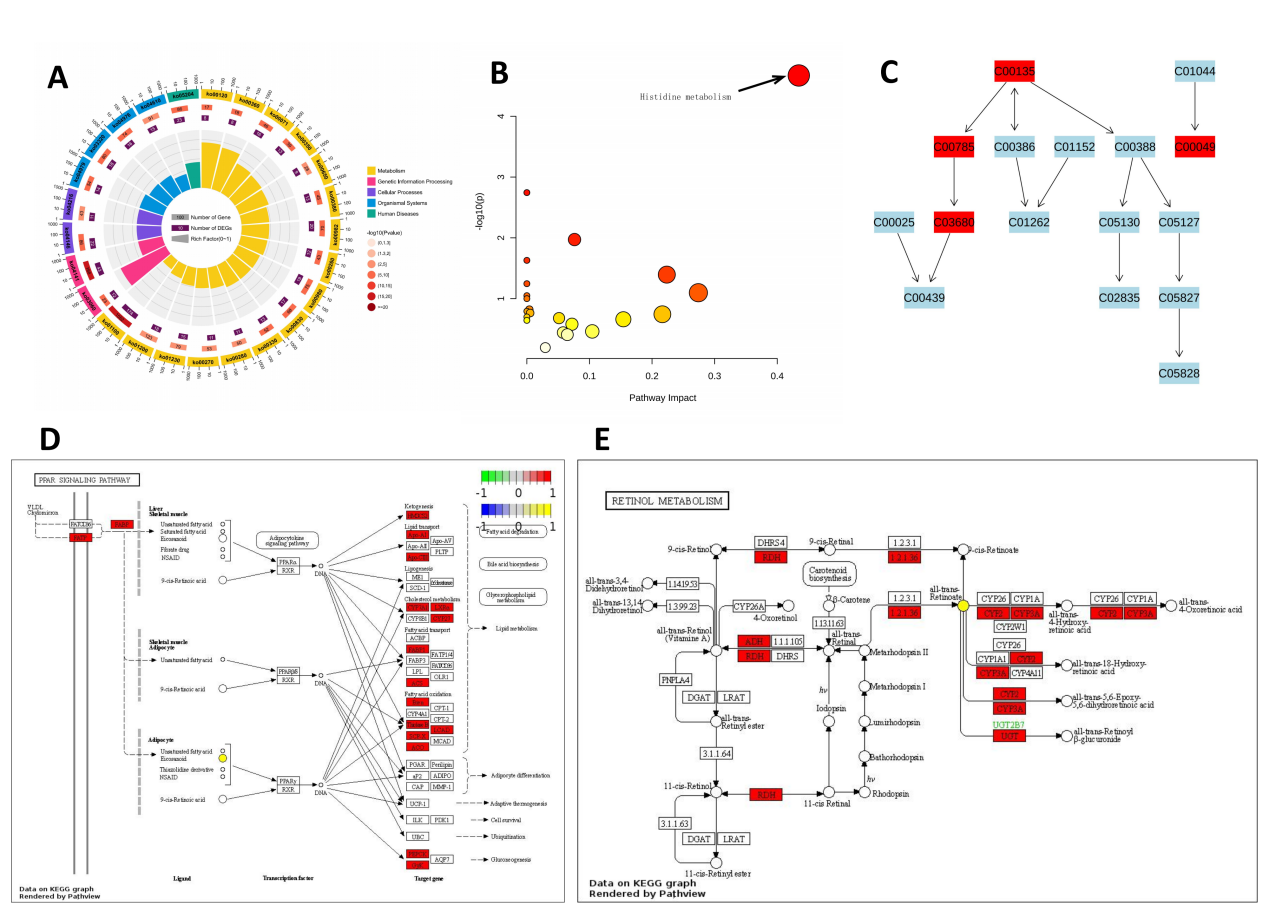
**

**Figure S5**

**
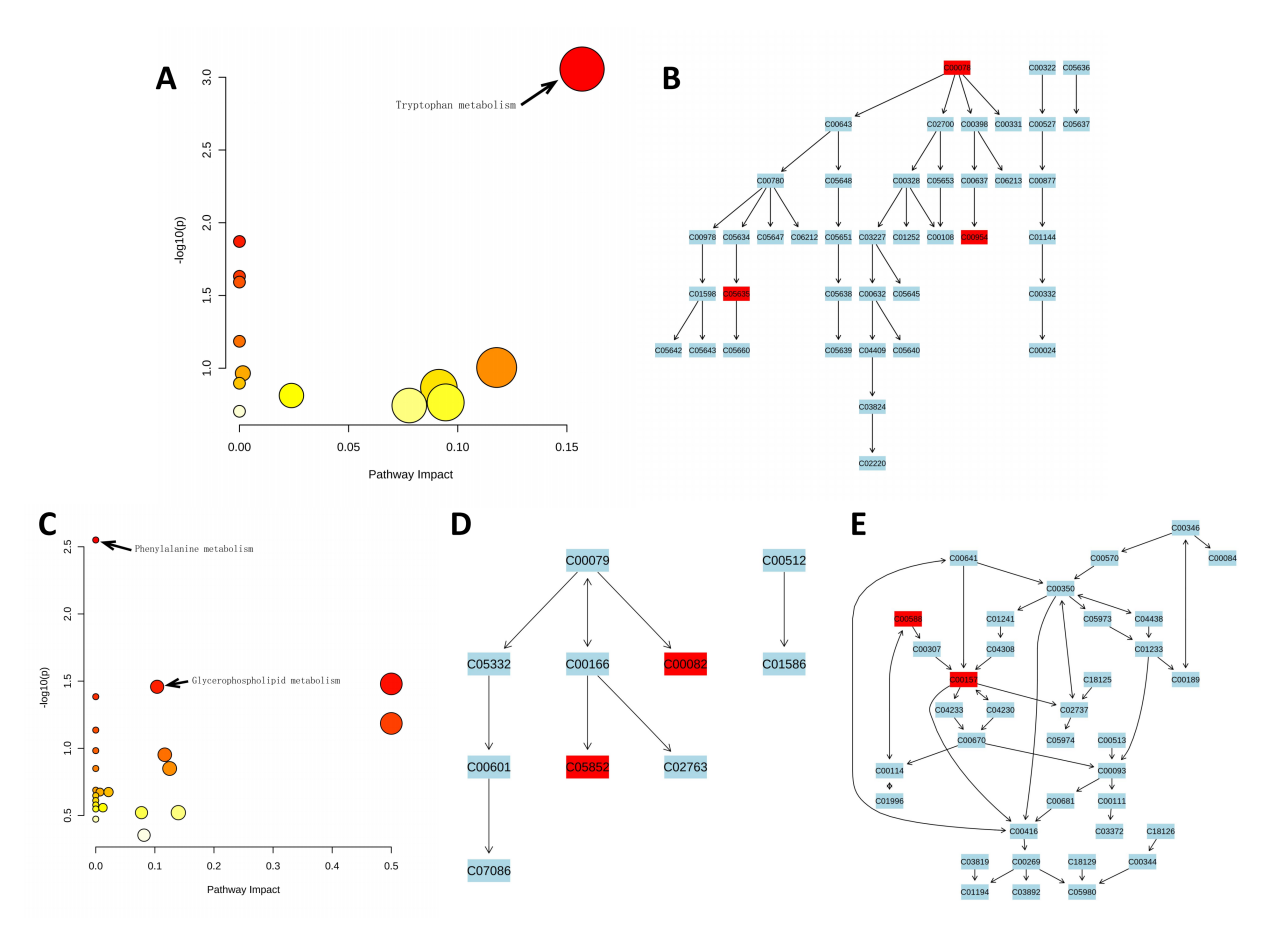
**

**Figure S6**

**
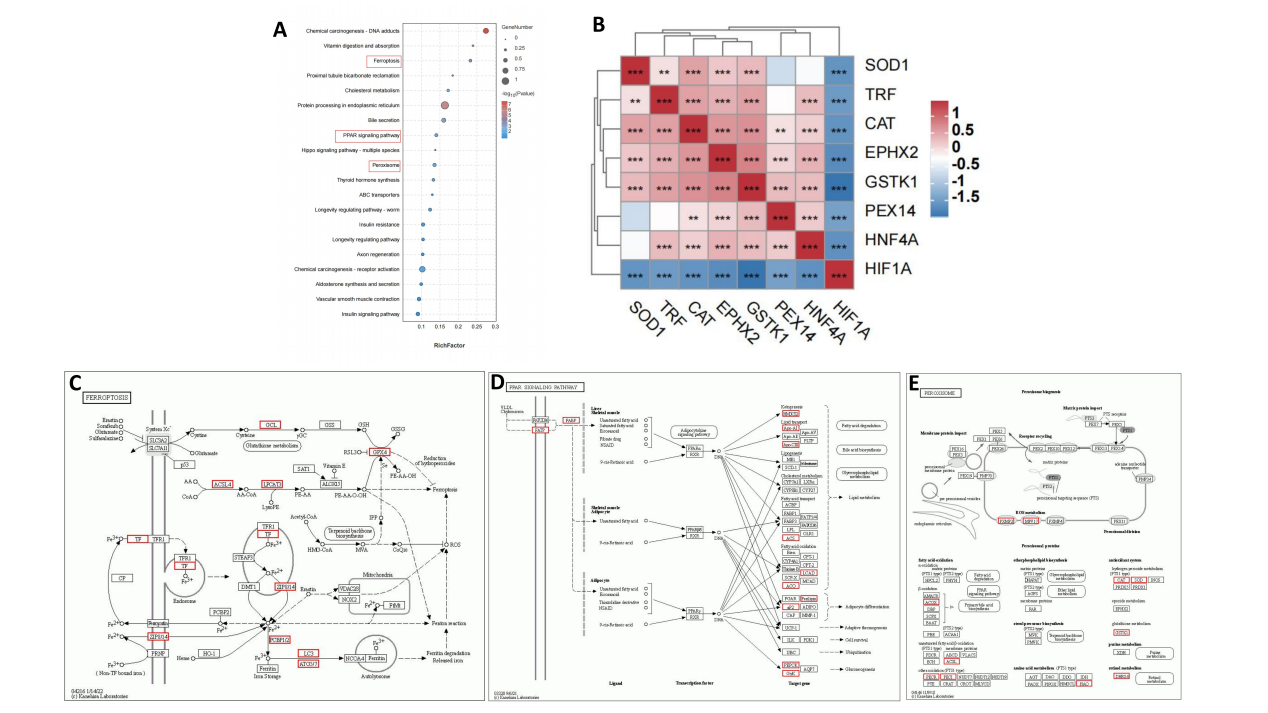
**
